# Supplementary material for: Prior expectations guide multisensory integration during face-to-face communication
Source: PLoS Comput Biol. 2025 Sep 12;21(9):e1013468. doi: 10.1371/journal.pcbi.1013468 (PMC12448992; doi:10.1371/journal.pcbi.1013468)
Supplement: S1 Text — (DOCX) [file pcbi.1013468.s001.docx]

# S1 text. Response times

In the present study, response times represented a measure of secondary interest that we evaluated in a supplementary analysis for completeness. Response times were expected to increase depending on task difficulty, which in turn may have increased due to three different factors. First, spatial localization was expected to be more difficult for the auditory than visual modality, in line with established evidence that spatial uncertainty is higher in audition than vision [1,2]. Second, we anticipated spatial localization to be more difficult at low AV spatial disparities, based on the notion that the uncertainty about the underlying causal structure (i.e. common versus separate sources) is higher when audiovisual stimuli are closer in space, thus rendering spatial localization more challenging in ventriloquist paradigms [3]. On the contrary, resolving the underlying causal structure is expected to be easier when stimuli are spatially congruent (i.e. no spatial disparity) or further apart in space (i.e. high spatial disparity). Third, we evaluated the possibility of a dual-task effect [4,5] for the communicative condition: in addition to spatial localization, participants had to attend to each word's meaning and infer the common theme communicated by the speaker, similar to real-life social interactions. Finally, we assessed additive or interactive effects between these three factors. For each trial, we measured the response times starting from the onset of the report cue. We averaged the participants’ median response times in each experimental condition and for each spatial disparity level and entered them into a 2 (action intention: communicative or non-communicative) × 2 (response modality: auditory or visual report) × 3 (spatial disparity: 0°: none; 9°: low; 18°: high) repeated measures ANOVA. Results are comprehensively displayed in Figure S1 and summarised in Tables S5 (descriptive statistics), S6 (ANOVA results) and S7 (post-hoc tests for significant interactions).

In Experiment 1, we found a significant main effect of response modality (p < 0.001, η^2^ = 0.22): participants were slower in reporting the auditory position compared to the visual position, confirming that spatial localization is more difficult in audition than vision [1,2]. Further, we found a main effect of spatial disparity (p = 0.004, η^2^ = 0.01): responses were slower at low AV spatial disparities, confirming that causal uncertainty impacts multisensory perceptual inference [3]. This was particularly the case for auditory localization, as indicated by a significant response modality × spatial disparity interaction (p < 0.001, η^2^ = 0.01). Hence, causal uncertainty was higher under increased perceptual uncertainty, in line with the principles of Bayesian Causal Inference [3]. Additionally, we found a significant action intention × response modality interaction (p = 0.002, η^2^ = 0.03): participants were slower in the non-communicative condition for auditory localization, while they were slower in the communicative condition for visual localization. However, please note that significant post-hoc comparisons reflected overall differences in response modality (i.e. auditory localization was slower than visual localization). Finally, there was a significant action intention × spatial disparity interaction (p < 0.001, η^2^ = 0.01): responses were slower at low spatial disparities for the communicative condition. Overall, these results suggest that perceptual uncertainty, causal uncertainty and dual-task costs interacted with each other to increase task difficulty.

In Experiment 2, we confirmed the significant main effects of response modality (p < 0.011, η^2^ = 0.02) and spatial disparity (p < 0.001, η^2^ = 0.01), and their interaction (p < 0.001, η^2^ = 0.01): during auditory localization, participants were faster at high AV spatial disparities. Moreover, we found a significant main effect of action intention (p < 0.001, η^2^ = 0.49): participants’ responses were slower in the communicative condition, in line with the presence of dual-task costs [4,5]. Plausibly, this effect arose only in Experiment 2 because we matched the stimuli duration (and thereby the associated perceptual uncertainty) across the two action intention conditions. Additionally, we found a significant action intention × response modality interaction (p < 0.001, η^2^ = 0.02): participants were slower when reporting the auditory than visual position in the communicative condition. Finally, there was a significant action intention × spatial disparity interaction (p = 0.03, η^2^ = 0.00): responses were slower at low spatial disparities in the communicative condition. Overall, we therefore confirmed that perceptual uncertainty, causal uncertainty and dual-task costs interacted with each other to increase task difficulty.

**References**

1. Alais D, Burr D. The Ventriloquist Effect Results from Near-Optimal Bimodal Integration. Current Biology. 2004;14: 257–262. doi:10.1016/j.cub.2004.01.029

2. Welch RB, Warren DH. Immediate Perceptual Response to Intersensory Discrepancy. 1980.

3. Ferrari A, Noppeney U. Attention controls multisensory perception via two distinct mechanisms at different levels of the cortical hierarchy. PLoS Biol. 2021;19(11):e3001465. Published 2021 Nov 18. doi:10.1371/journal.pbio.3001465

4. Pashler H. Dual-Task Interference in Simple Tasks: Data and Theory. 1994.

5. Sigman M, Dehaene S. Dynamics of the Central Bottleneck: Dual-Task and Task Uncertainty. Fahle M, editor. PLoS Biol. 2006;4: e220. doi:10.1371/journal.pbio.0040220
