# Supplementary figures and images for: Prior expectations guide multisensory integration during face-to-face communication
Source: PLoS Comput Biol. 2025 Sep 12;21(9):e1013468. doi: 10.1371/journal.pcbi.1013468 (PMC12448992; doi:10.1371/journal.pcbi.1013468)

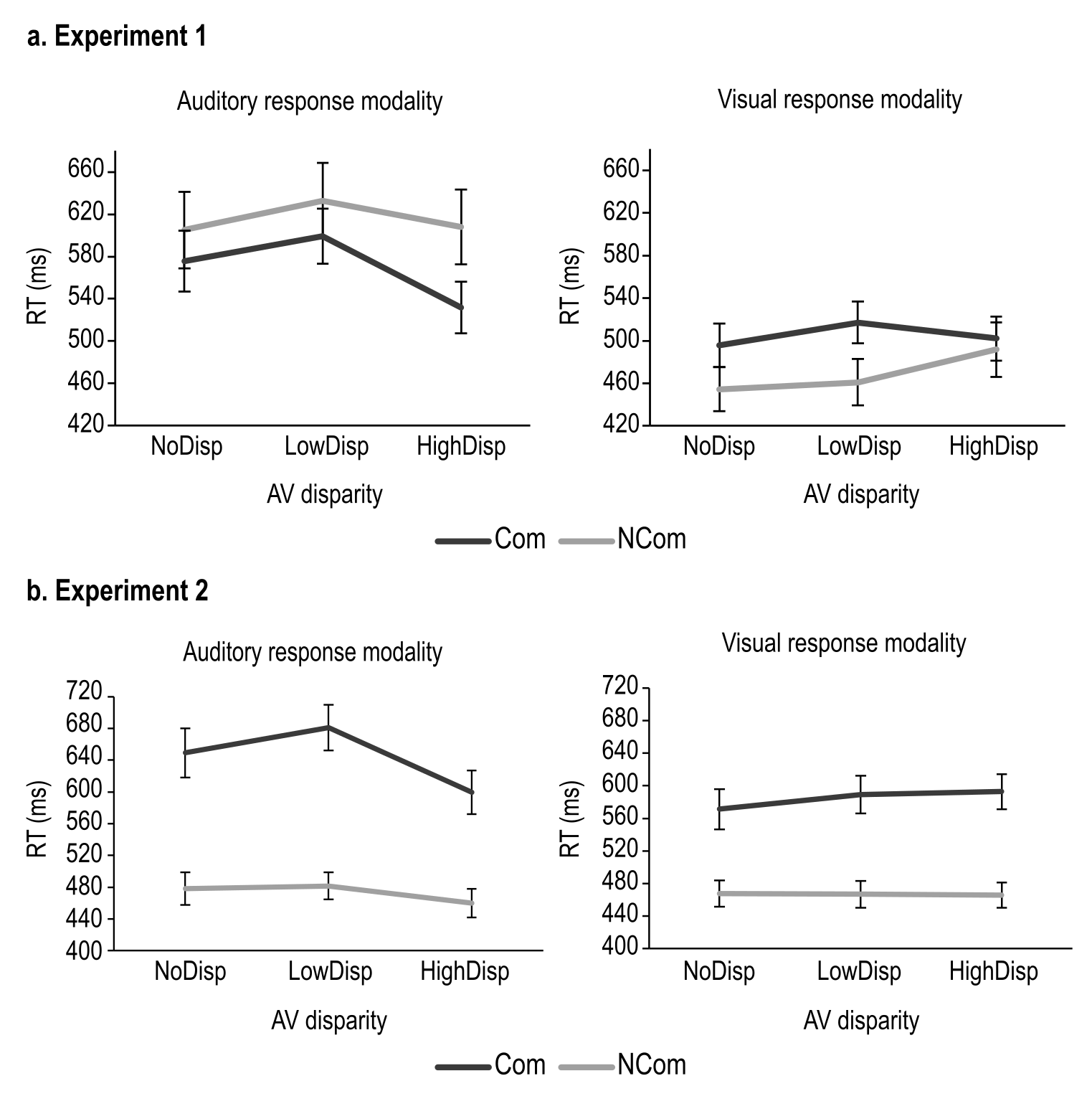

Supplement: S1 Fig — Across-participants’ mean (± SEM) response times in Experiments 1 and 2, as a function of response modality (auditory; visual), action intention (communicative: Com; non-communicative: NCom) and spatial disparity (0°: NoDisp; 9°: LowDisp; 18°: HighDisp). (TIF) [file pcbi.1013468.s002.tif]

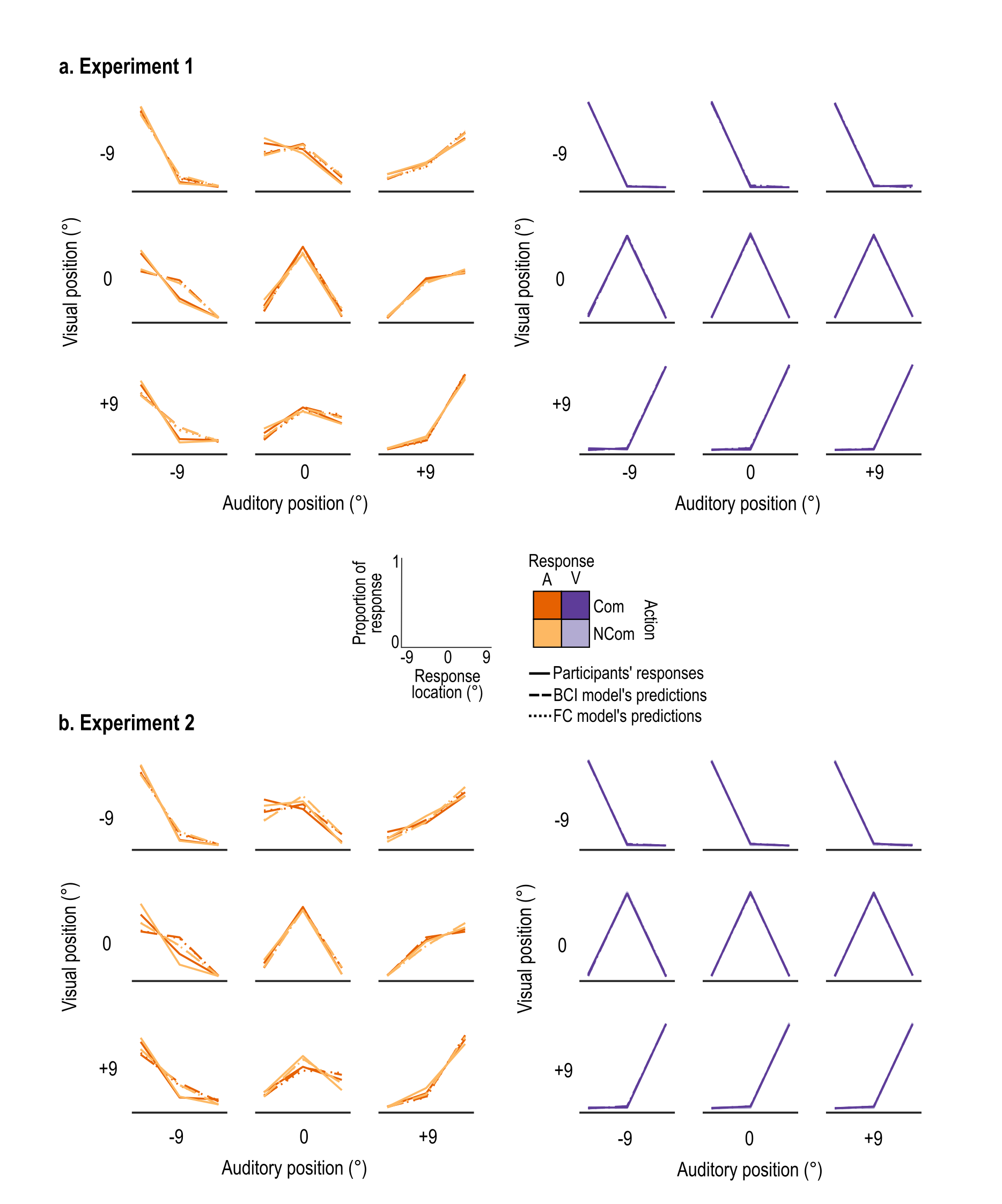

Supplement: S2 Fig — Distributions of spatial estimates in Experiments 1 and 2 given by participants’ localization responses (solid lines) or predicted by the BCI Separated model (dashed line) and FC Separated model (dotted line) fitted to each participant’s responses as a function of response modality (auditory: A; visual: V), action intention (communicative: Com; non-communicative: NCom) and stimuli position (0, 9, 18 degrees visual angle). In the 3 × 3 subplots, the visual position is represented on the y-axis and the auditory position is represented on the x-axis. For each audiovisual combination (each subplot), the 3 possible answers are represented on the x-axis (left, centre, right) and participants’ proportion of responses for each of the possible answers is represented on the y-axis. (TIF) [file pcbi.1013468.s003.tif]

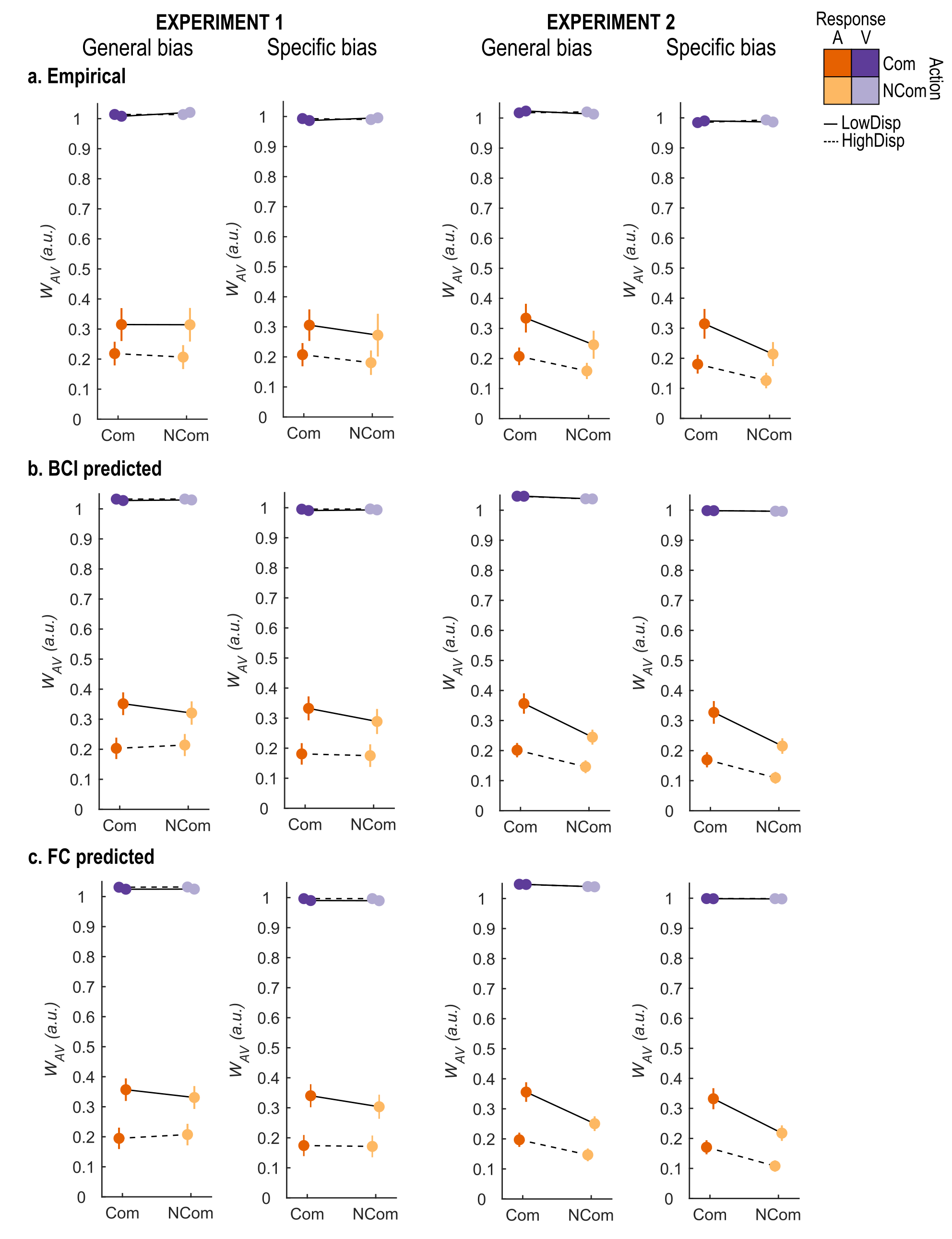

Supplement: S3 Fig — a) Across participants’ mean ± SEM empirical wAV obtained from the Experiments, b) predicted wAV obtained from model predictions of the BCI Separated model and c) predicted wAV obtained from model predictions of the FC Separated model. Results are plotted as a function of action intention (communicative: Com; non-communicative: NCom), response modality (auditory: A; visual: V) and audiovisual spatial disparity (9°: LowDisp; 18°: HighDisp). An index equal to 1 (respectively, 0) indicates pure visual (respectively, auditory) influence on participants’ localization responses; values between 0 and 1 indicate intermediate degrees of audiovisual integration. General bias: wAV obtained using the reported location in audiovisual congruent trials averaged across participants and experimental conditions (action intention × response modality). Specific bias: wAV obtained using the participant-specific and condition-specific average reported location in audiovisual congruent trials. (TIF) [file pcbi.1013468.s004.tif]

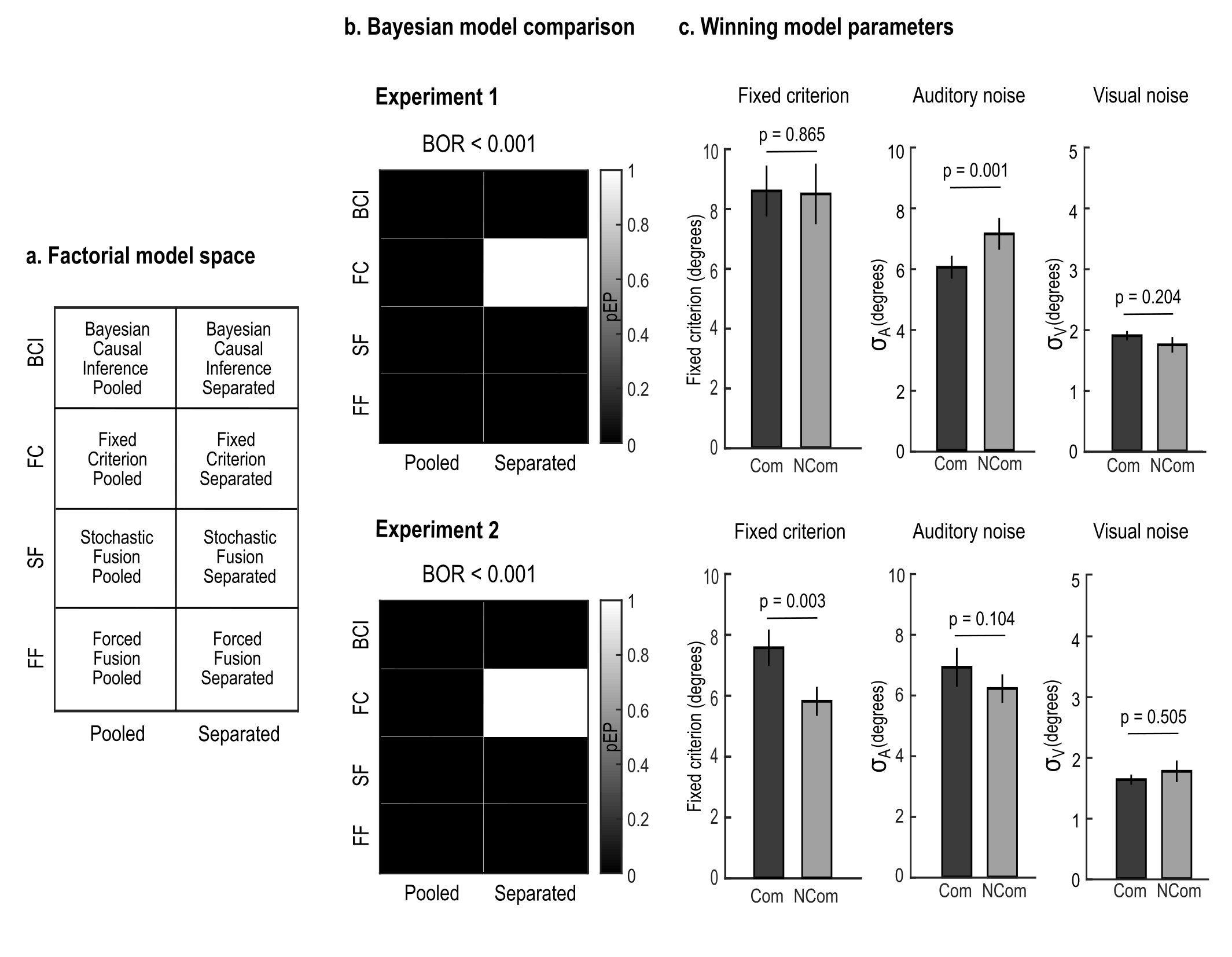

Supplement: S4 Fig — a) To determine whether non-optimal decision strategies best explained each participant’s localisation responses, and to evaluate the modulatory influence of action intention (communicative vs. non-communicative), we performed Bayesian model comparison in a 4 (BCI vs FC vs SF vs FF) × 2 (Pooled vs Separated action intention conditions) factorial model space. b) Protected exceedance probability (pEP, grayscale) of each model in the 4 × 2 model comparison factorial space, i.e., how likely each model is to explain the data compared to the other models. Bayesian Omnibus Risk (BOR) represents the probability that the results are due to chance. c) Across participants’ mean ± SEM parameter estimates of fixed criterion (kC), auditory noise (σA), and visual noise (σV) of the winning model (i.e., FC Separated) as a function of action intention. p-values based on two-tailed between-subject permutation tests. Top and bottom rows show the results of Experiment 1 and 2 respectively. (TIF) [file pcbi.1013468.s005.tif]

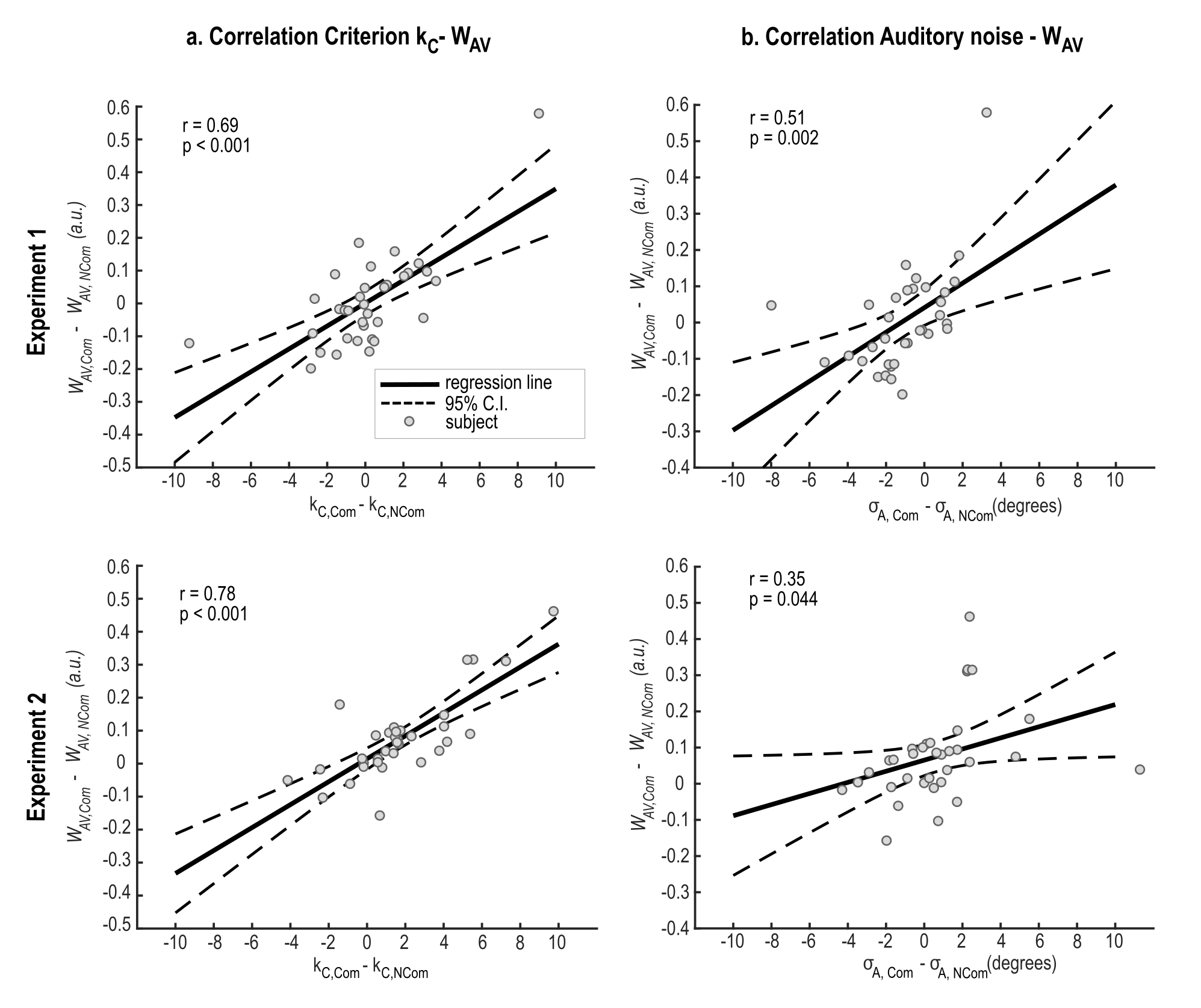

Supplement: S5 Fig — Across participants’ correlation between increases of wAV for the communicative relative to the non-communicative condition (wAV ,Com − wAV,NCom) and the respective effect on a) the common cause prior (kC,Com − kC,NCom) and b) the auditory noise (𝜎A, Com − 𝜎A, NCom) of the winning model (FC Separated). Pearson’s correlation coefficients (r) and p-values (p) obtained from the two linear regression models: (1) wAV = β₀ + β₁×kC + ε and (2) wAV = β₀ + β₁× 𝜎A + ε, where β₀= intercept, β₁= slope coefficient, ε = residual error. Thick black line and dashed lines respectively show the linear regression line and the 95% confidence intervals (C.I.) from the linear regression models. Grey dots represent individual participants. Top and bottom rows show the results of Experiments 1 and 2 respectively. (TIF) [file pcbi.1013468.s006.tif]
