# Supplementary material for: Prior expectations guide multisensory integration during face-to-face communication
Source: PLoS Comput Biol. 2025 Sep 12;21(9):e1013468. doi: 10.1371/journal.pcbi.1013468 (PMC12448992; doi:10.1371/journal.pcbi.1013468)
Supplement: S1 Table — Across-participants’ mean (± SEM) wAV as a function of action intention (communicative: Com; non-communicative: NCom), response modality (repA: auditory; repV: visual) and audiovisual spatial disparity (9°: LowDisp; 18°: HighDisp) for Experiments 1 and 2. (DOCX) [file pcbi.1013468.s007.docx]

# S1 Table. Audiovisual weight index (*w_AV_*): descriptive statistics

| *w_AV_* (a.u.) | ComRepA | NComRepA | ComRepV | NComRepV |
| --- | --- | --- | --- | --- |
| Experiment 1 |  |  |  |  |
| LowDisp | 0.31 (±0.05) | 0.31 (±0.06) | 1.01 (±0.00) | 1.02 (±0.00) |
| HighDisp | 0.22 (±0.04) | 0.21 (±0.04) | 1.01 (±0.00) | 1.01 (±0.00) |
| Experiment 2 |  |  |  |  |
| LowDisp | 0.33 (±0.05) | 0.24 (±0.05) | 1.03 (±0.00) | 1.02 (±0.00) |
| HighDisp | 0.21 (±0.03) | 0.16 (±0.03) | 1.02 (±0.00) | 1.03 (±0.00) |

Across-participants' mean (± SEM) ***w_AV_*** as a function of action intention (communicative: Com; non-communicative: NCom), response modality (repA: auditory; repV: visual) and audiovisual spatial disparity (9°: LowDisp; 18°: HighDisp) for Experiments 1 and 2.
