# Supplementary material for: Prior expectations guide multisensory integration during face-to-face communication
Source: PLoS Comput Biol. 2025 Sep 12;21(9):e1013468. doi: 10.1371/journal.pcbi.1013468 (PMC12448992; doi:10.1371/journal.pcbi.1013468)
Supplement: S2 Table — Across participants’ mean (±SEM) of the models’ parameters: pcommon, common-cause prior probability; kC, fixed criterion (° visual angle); η, probability of fusion response; σP, spatial prior standard deviation (° visual angle); σA, auditory likelihood standard deviation (° visual angle); σV, visual likelihood standard deviation (° visual angle). Model architectures: Bayesian Causal Inference (BCI); Fixed Criterion (FC); Stochastic Fusion (SF); Forced Fusion (FF). While “Pooled” models do not account for the influence of action intention (communicative: Com vs. non-communicative: NCom), “Separated” models have separate parameters for each action intention condition. (DOCX) [file pcbi.1013468.s008.docx]

# S2 Table. Bayesian models parameters

| **Model** | $p_{common}$ | $k_{C}$ | $\eta$ | $\sigma_{P}$ | $\sigma_{A}$ | $\sigma_{V}$ |
| --- | --- | --- | --- | --- | --- | --- |
| **Experiment 1** | |  |  |  |  |  |
| BCI  Pooled | 0.33  (±0.04) | n/a | n/a | 23.98  (±1.48) | 7.21  (±0.78) | 1.96  (±0.10) |
| BCI  Separated | Com:  0.38  (±0.04)  NCom:  0.29  (±0.04) | n/a | n/a | Com:  22.51  (±1.70)  NCom:  24.38  (±1.43) | Com:  6.79 (±0.79)  NCom:  7.60 (±0.80) | Com:  1.91 (±0.08)  NCom:  1.81 (±0.14) |
| FC Pooled | n/a | 8.46 (±0.89) | n/a | 18.53 (±1.85) | 6.70 (±0.45) | 1.91 (±0.09) |
| FC Separated | n/a | Com:  8.58  (±0.85)  NCom:  8.49  (±1.01) | n/a | Com:  16.20  (±1.71)  NCom:  18.92  (±1.78) | Com:  5.95  (±0.37)  NCom:  7.04  (±0.52) | Com:  1.92  (±0.08)  NCom:  1.77  (±0.13) |
| SF Pooled | n/a | n/a | 0.16 (±0.02) | 22.21 (±1.46) | 7.79 (±0.77) | 1.77 (±0.13) |
| SF  Separated | n/a | n/a | Com:  0.14  (±0.02)  NCom:  0.16  (±0.03) | Com:  20.50  (±1.44)  NCom:  22.23  (±1.44) | Com:  7.61  (±0.77)  NCom:  7.59  (±0.57) | Com:  1.83  (±0.11)  NCom:  1.62  (±0.18) |
| FF Pooled | n/a | n/a | n/a | 28.75  (±0.82) | 10.29  (±0.40) | 7.61  (±0.42) |
| FF  Separated | n/a | n/a | n/a | Com:  29.06  (±0.67)  NCom:  28.12  (±1.04) | Com:  9.82  (±0.40)  NCom:  10.56  (±0.39) | Com:  7.25  (±0.42)  NCom:  7.81  (±0.45) |
| **Experiment 2** | |  |  |  |  |  |
| BCI  Pooled | 0.29  (±0.03) | n/a | n/a | 21.32  (±1.63) | 6.67  (±0.44) | 1.78  (±0.12) |
| BCI  Separated | Com:  0.34  (±0.04)  NCom:  0.22  (±0.03) | n/a | n/a | Com:  21.70  (±1.64)  NCom:  20.72  (±1.67) | Com:  6.81 (±0.47)  NCom:  6.28 (±0.45) | Com:  1.41 (±0.14)  NCom:  1.73 (±0.19) |
| FC Pooled | n/a | 6.84 (±0.48) | n/a | 14.42 (±1.54) | 6.70 (±0.52) | 1.79 (±0.11) |
| FC Separated | n/a | Com:  7.57  (±0.60)  NCom:  5.81  (±0.48) | n/a | Com:  14.72  (±1.65)  NCom:  15.30  (±1.68) | Com:  6.97  (±0.65)  NCom:  6.26  (±0.47) | Com:  1.60  (±0.08)  NCom:  1.74  (±0.18) |
| SF Pooled | n/a | n/a | 0.12 (±0.01) | 19.80 (±1.51) | 7.55 (±0.60) | 1.55 (±0.17) |
| SF  Separated | n/a | n/a | Com:  0.14  (±0.01)  NCom:  0.15  (±0.01) | Com:  20.55  (±1.53)  NCom:  18.81  (±1.51) | Com:  8.34  (±0.71)  NCom:  6.78  (±0.58) | Com:  1.32  (±0.15)  NCom:  1.58  (±0.20) |
| FF Pooled | n/a | n/a | n/a | 28.80  (±0.64) | 10.13  (±0.32) | 7.47  (±0.24) |
| FF  Separated | n/a | n/a | n/a | Com:  28.50  (±0.82)  NCom:  29.39  (±0.40) | Com:  10.41  (±0.39)  NCom:  9.88  (±0.29) | Com:  7.39  (±0.28)  NCom:  7.67  (±0.26) |

Across participants' mean (±SEM) of the models’ parameters: $p_{common}$, common-cause prior probability; $k_{C}$, fixed criterion (° visual angle); $\eta$, probability of fusion response; $\sigma_{P}$, spatial prior standard deviation (° visual angle); $\sigma_{A}$, auditory likelihood standard deviation (° visual angle); $\sigma_{V}$, visual likelihood standard deviation (° visual angle). Model architectures: Bayesian Causal Inference (BCI); Fixed Criterion (FC); Stochastic Fusion (SF); Forced Fusion (FF). While “Pooled” models do not account for the influence of action intention (communicative: Com vs. non-communicative: NCom), “Separated” models have separate parameters for each action intention condition.
