# Supplementary material for: Prior expectations guide multisensory integration during face-to-face communication
Source: PLoS Comput Biol. 2025 Sep 12;21(9):e1013468. doi: 10.1371/journal.pcbi.1013468 (PMC12448992; doi:10.1371/journal.pcbi.1013468)
Supplement: S3 Table — Model architectures: Bayesian Causal Inference (BCI); Forced Fusion (FF). To determine which model architecture best explained each participant’s localisation responses, and to evaluate the modulatory influence of action intention (communicative vs. non-communicative), we performed random-effect Bayesian model comparison in a 2 (BCI vs FF) × 2 (“Pooled” action intention conditions vs “Separated” conditions) factorial model space. We report across participants’ mean (±SEM) coefficient of determination (R2) and protected exceedance probability (pEP; probability that a model is more likely than the other models, beyond differences due to chance). (DOCX) [file pcbi.1013468.s009.docx]

# S3 Table. Bayesian model comparison (4 models)

| **Model** | BCI Pooled | BCI Separated | FF Pooled | FF Separated |
| --- | --- | --- | --- | --- |
| **Experiment 1** |  |  |  |  |
| $R^{2}$ | 0.85 (±0.01) | 0.95 (±0.01) | 0.52 (±0.02) | 0.86 (±0.01) |
| $pEP$ | 0 | 1 | 0 | 0 |
| **Experiment 2** |  |  |  |  |
| $R^{2}$ | 0.85 (±0.01) | 0.95 (±0.01) | 0.51 (±0.01) | 0.85 (±0.01) |
| $pEP$ | 0 | 1 | 0 | 0 |

Model architectures: Bayesian Causal Inference (BCI); Forced Fusion (FF). To determine which model architecture best explained each participant’s localisation responses, and to evaluate the modulatory influence of action intention (communicative vs. non-communicative), we performed random-effect Bayesian model comparison in a 2 (BCI vs FF) × 2 (“Pooled” action intention conditions vs “Separated” conditions) factorial model space. We report across participants’ mean (±SEM) coefficient of determination ($R^{2}$) and protected exceedance probability ($pEP$; probability that a model is more likely than the other models, beyond differences due to chance).
