# Supplementary material for: Prior expectations guide multisensory integration during face-to-face communication
Source: PLoS Comput Biol. 2025 Sep 12;21(9):e1013468. doi: 10.1371/journal.pcbi.1013468 (PMC12448992; doi:10.1371/journal.pcbi.1013468)
Supplement: S5 Table — Across participants’ mean (±SEM) response times as a function of action intention (communicative: Com; non-communicative: NCom), response modality (repA: auditory; repV: visual) and audiovisual spatial disparity (0°: NoDisp; 9°: LowDisp; 18°: HighDisp) for Experiments 1 and 2. (DOCX) [file pcbi.1013468.s011.docx]

# S5 Table. Response times: descriptive statistics

| **RT (ms)** | **ComRepA** | **NComRepA** | **ComRepV** | **NComRepV** |
| --- | --- | --- | --- | --- |
| **Experiment 1** |  |  |  |  |
| NoDisp | 575.495 (±28.84) | 605.06 (±36.35) | 495.66 (±20.26) | 454.37 (±20.74) |
| LowDisp | 599.13 (±26.06) | 632.80 (±35.78) | 517.02 (±19.64) | 460.90 (±21.99) |
| HighDisp | 532.00 (±23.49) | 608.03 (±35.33) | 502.88 (±20.78) | 491.67 (±25.74) |
| **Experiment 2** |  |  |  |  |
| NoDisp | 649.16 (±31.05) | 478.24 (±20.30) | 571.19 (±24.27) | 467.33 (±16.25) |
| LowDisp | 680.79 (±28.85) | 481.49 (±17.14) | 589.03 (±23.12) | 466.65 (±16.68) |
| HighDisp | 599.55 (±27.51) | 459.93 (±18.33) | 592.89 (±21.16) | 465.99 (±15.58) |

Across participants’ mean (±SEM) response times as a function of action intention (communicative: Com; non-communicative: NCom), response modality (repA: auditory; repV: visual) and audiovisual spatial disparity (0°: NoDisp; 9°: LowDisp; 18°: HighDisp) for Experiments 1 and 2.
