# Supplementary material for: Prior expectations guide multisensory integration during face-to-face communication
Source: PLoS Comput Biol. 2025 Sep 12;21(9):e1013468. doi: 10.1371/journal.pcbi.1013468 (PMC12448992; doi:10.1371/journal.pcbi.1013468)
Supplement: S6 Table — Main effects and interactions for the response times in the 2 (action intention: communicative; non-communicative) × 2 (response modality: auditory; visual) × 3 (spatial disparity: none; low; high) repeated measures ANOVA. Greenhouse-Geisser correction is applied in case of violation of sphericity (Mauchly’s test). (DOCX) [file pcbi.1013468.s012.docx]

# S6 Table. Response times: ANOVA results

|  | Experiment 1 | | | Experiment 2 | | |
| --- | --- | --- | --- | --- | --- | --- |
|  | **F-value**  **(df1, df2)** | **p-value** | **Effect size (η^2^)** | **F-value**  **(df1, df2)** | **p-value** | **Effect size (η^2^)** |
| Act | 0.04  (1, 33) | 0.835 | 0.00 | 98.36  (1, 33) | **< .001** | 0.49 |
| Resp | 72.12  (1, 33) | **< .001** | 0.22 | 7.19  (1,33) | **0.011** | 0.02 |
| Disp | 7.18  (1.50, 49.55) | **0.004** | 0.01 | 11.88  (1.48, 48.90) | **< .001** | 0.01 |
| Act×Resp | 10.99  (1, 33) | **0.002** | 0.03 | 15.14  (1, 33) | **< .001** | 0.02 |
| Act×Disp | 10.83  (1.77, 58.65) | **< .001** | 0.01 | 3.78  (1.69, 55.70) | **0.03** | 0.00 |
| Resp×Disp | 10.46  (1.88, 62.04) | **< .001** | 0.01 | 11.74  (1.75, 57.93) | **< .001** | 0.01 |
| Act×Resp×Disp | 0.56  (1.92, 63.23) | 0.566 | 0.00 | 5.97  (1.93, 63.60) | **0.005** | 0.00 |

Main effects and interactions for the response times in the 2 (action intention: communicative; non-communicative) × 2 (response modality: auditory; visual) × 3 (spatial disparity: none; low; high) repeated measures ANOVA. Greenhouse-Geisser correction is applied in case of violation of sphericity (Mauchly's test).
