# Supplementary material for: Prior expectations guide multisensory integration during face-to-face communication
Source: PLoS Comput Biol. 2025 Sep 12;21(9):e1013468. doi: 10.1371/journal.pcbi.1013468 (PMC12448992; doi:10.1371/journal.pcbi.1013468)
Supplement: S7 Table — Post-hoc comparisons for the significant two-way interactions of the response times ANOVA: Action Intention × Response Modality; Action Intention × Spatial Disparity; Response Modality × Spatial Disparity. Com: communicative action; NCom: non-communicative action; Aud: auditory response; Vis: visual response; NoDisp: no spatial disparity (0°); LowDisp: low disparity (9°); HighDisp: high disparity (18°). P-values were adjusted using the Holm correction. (DOCX) [file pcbi.1013468.s013.docx]

# S7 Table. Response times: post-hoc comparisons

|  |  | Experiment 1 | | | Experiment 2 | | |
| --- | --- | --- | --- | --- | --- | --- | --- |
|  |  | **Mean Difference**  **(SE)** | **t** | **p_Holm_** | **Mean Difference**  **(SE)** | **t** | **p_Holm_** |
| **Act×Resp** | |  |  |  |  |  |  |
| Com, Aud | NCom, Aud | -46.42  (27.35) | -1.679 | 0.192 | 169.95  (15.98) | 10.635 | **< .001** |
|  | Com, Vis | 63.69  (17.55) | 3.628 | **0.002** | 58.80  (13.91) | 4.226 | **< .001** |
| NCom, Aud | NCom, Vis | 146.31  (17.55) | 8.335 | **< .001** | 6.57  (13.91) | 0.472 | 0.639 |
| Com, Vis | NCom, Vis | 36.21  (27.35) | 1.324 | 0.192 | 117.71  (15.98) | 7.3660 | **< .001** |
| **Act×Disp** | | |  |  |  |  |  |
| Com, NoDisp | NCom, NoDisp | 5.86  (25.05) | 0.234 | 1.000 | 137.39  (15.79) | 8.701 | **< .001** |
|  | Com, LowDisp | -22.50  (7.80) | -2.884 | 0.064 | -24.73  (7.44) | -3.324 | **0.006** |
|  | Com, HighDisp | 18.14  (7.80) | 2.325 | 0.259 | 13.95  (7.44) | 1.875 | 0.252 |
| NCom, NoDisp | NCom, LowDisp | -17.13  (7.80) | -2.196 | 0.328 | -1.28  (7.44) | -0.172 | 0.863 |
|  | NCom, HighDisp | -20.14  (7.80) | -2.581 | 0.142 | 9.83  (7.44) | 1.321 | 0.413 |
| Com, LowDisp | NCom, LowDisp | 11.23  (25.05) | 0.448 | 1.000 | 160.84  (15.79) | 10.186 | **< .001** |
|  | Com, HighDisp | 40.63  (7.80) | 5.209 | **< .001** | 38.69  (7.44) | 5.200 | **< .001** |
| NCom, LowDisp | NCom, HighDisp | -3.00  (7.80) | -0.385 | 1.000 | 11.11  (7.44) | 1.493 | 0.413 |
| Com, HighDisp | NCom, HighDisp | -32.41  (25.05) | -1.294 | 1.000 | 133.26  (15.79) | 8.440 | **< .001** |
| **Resp×Disp** | |  |  |  |  |  |  |
| Aud, NoDisp | Aud, LowDisp | -25.69  (8.58) | -2.993 | **0.017** | -17.44  (7.78) | -2.243 | 0.186 |
|  | Aud, HighDisp | 20.28  (8.58) | 2.361 | 0.059 | 33.96  (7.78) | 4.368 | **< .001** |
| Vis, NoDisp | Vis, LowDisp | -13.944  (8.58) | -1.625 | 0.213 | -8.58  (7.78) | -1.103 | 1.000 |
|  | Vis, HighDisp | -22.264  (8.58) | -2.594 | **0.042** | -10.18  (7.78) | -1.309 | 1.000 |
| Aud, LowDisp | Aud, HighDisp | 45.953  (8.58) | 5.354 | **< .001** | 51.40  (7.78) | 6.611 | **< .001** |
| Vis, LowDisp | Vis, HighDisp | -8.320  (8.58) | -0.969 | 0.334 | -1.60  (7.78) | -0.206 | 1.000 |

Post-hoc comparisons for the significant two-way interactions of the response times ANOVA: Action Intention × Response Modality; Action Intention × Spatial Disparity; Response Modality × Spatial Disparity. Com: communicative action; NCom: non-communicative action; Aud: auditory response; Vis: visual response; NoDisp: no spatial disparity (0°); LowDisp: low disparity (9°); HighDisp: high disparity (18°). P-values were adjusted using the Holm correction.
