# Supplementary material for: Prior expectations guide multisensory integration during face-to-face communication
Source: PLoS Comput Biol. 2025 Sep 12;21(9):e1013468. doi: 10.1371/journal.pcbi.1013468 (PMC12448992; doi:10.1371/journal.pcbi.1013468)
Supplement: S9 Table — Main effects and interactions for the audiovisual weight index (wAV) in Experiments 1 and 2 in the 2 (action intention: communicative vs. non-communicative) × 2 (response modality: auditory vs. visual) × 2 (audiovisual disparity: low vs. high) factorial design. The wAV was computed using the average reported location for congruent trials specific for each participant in the four experimental conditions (Action × Response modality). P-values are based on two tailed permutation tests. Effect sizes [95% CI] correspond to the difference of the across participants’ mean empirical effect and the mean of the non-parametric null-distribution. (DOCX) [file pcbi.1013468.s015.docx]

# S9 Table. Audiovisual weight index (*w_AV_*) sensitivity analysis: statistical significance

|  | Experiment 1 | | | Experiment 2 | | |
| --- | --- | --- | --- | --- | --- | --- |
|  | **p-value** | **Effect size** | **95% C.I.** | **p-value** | **Effect size** | **95% C.I.** |
| Act | 0.414 | -0.003 | [-0.02, 0.02] | 0.079 | 0.032 | [0.01, 0.06] |
| Resp | **< 0.001** | 0.839 | [0.81, 0.87] | **< 0.001** | 0.818 | [0.78, 0.86] |
| Disp | **< 0.001** | -0.047 | [-0.06, -0.04] | **< 0.001** | -0.048 | [-0.06, -0.03] |
| Act×Resp | 0.202 | -0.012 | [-0.04, 0.02] | **0.039** | -0.073 | [-0.11, -0.03] |
| Act×Disp | 0.914 | 0.006 | [-0.02, 0.03] | 0.164 | -0.020 | [-0.04, 0.00] |
| Resp×Disp | **0.004** | 0.105 | [0.08, 0.13] | **< 0.001** | 0.071 | [0.04, 0.10] |
| Act×Resp×Disp | 0.582 | 0.021 | [-0.02, 0.06] | 0.427 | -0.025 | [-0.08, 0.02] |

Main effects and interactions for the audiovisual weight index (*w_AV_*) in Experiments 1 and 2 in the 2 (action intention: communicative vs. non-communicative) × 2 (response modality: auditory vs. visual) × 2 (audiovisual disparity: low vs. high) factorial design. The *w_AV_* was computed using the average reported location for congruent trials specific to each participant in the four experimental conditions (Action × Response modality). P-values are based on two-tailed permutation tests. Effect sizes [95% CI] correspond to the difference between the across-participants' mean empirical effect and the mean of the non-parametric null distribution.
