# Supplementary material for: Prior expectations guide multisensory integration during face-to-face communication
Source: PLoS Comput Biol. 2025 Sep 12;21(9):e1013468. doi: 10.1371/journal.pcbi.1013468 (PMC12448992; doi:10.1371/journal.pcbi.1013468)
Supplement: Attachment — Submitted filename: Mazzi_Ferrari_response_reviewers.docx [file pcbi.1013468.s016.docx]

**Response to reviewers**

We are grateful to the reviewers and the editor for their thoughtful and constructive feedback. We have revised the manuscript thoroughly and responded to each point to the best of our ability. We hope that our changes and clarifications effectively address the concerns raised. Below, we have attached a copy of the reviews with a point-by-point reply to each issue raised; our replies are in blue. All additions to the manuscript are explained here, along with the corresponding page numbers. Additionally, all changes are highlighted in the manuscript using track changes.

**Journal Requirements**

1) We ask that a manuscript source file is provided at Revision. Please upload your manuscript file as a .doc, .docx, .rtf or .tex. If you are providing a .tex file, please upload it under the item type u2018LaTeX Source Fileu2019 and leave your .pdf version as the item type u2018Manuscriptu2019.

We have uploaded the manuscript as a source file (Word document).

2) We do not publish any copyright or trademark symbols that usually accompany proprietary names, eg ©, ®, or TM (e.g. next to drug or reagent names). Therefore please remove all instances of trademark/copyright symbols throughout the text, including: - ® on page: 8.

We have removed all instances of trademark/copyright symbols throughout the text.

3) Please upload all main figures as separate Figure files in .tif or .eps format. For more information about how to convert and format your figure files please see our guidelines:
https://journals.plos.org/ploscompbiol/s/figures

We have uploaded all main figures as .tiff files, formatted in accordance with the journal's guidelines.

4) We have noticed that you have uploaded Supporting Information files, but you have not included a list of legends. Please add a full list of legends for your Supporting Information files after the references list.

We have added legends for all Supporting Information files after the references list.

5) Figure 1 includes an image of an identifiable person. Please provide written confirmation or release forms, signed by the subject(s) (or their guardian), giving permission to be photographed and to have their images published under a Creative Commons license. You may upload permission forms to your submission file inventory as item type 'Other'. Otherwise, we kindly request that you remove the photograph.

We have provided written confirmation, signed by the subject, granting permission to be photographed and to have their images published under a Creative Commons license. The permission form has been uploaded to the submission files.

6) Some material included in your submission may be copyrighted. According to PLOSu2019s copyright policy, authors who use figures or other material (e.g., graphics, clipart, maps) from another author or copyright holder must demonstrate or obtain permission to publish this material under the Creative Commons Attribution 4.0 International (CC BY 4.0) License used by PLOS journals. Please closely review the details of PLOSu2019s copyright requirements here: PLOS Licenses and Copyright. If you need to request permissions from a copyright holder, you may use PLOS's Copyright Content Permission form.

Please respond directly to this email and provide any known details concerning your material's license terms and permissions required for reuse, even if you have not yet obtained copyright permissions or are unsure of your material's copyright compatibility. Once you have responded and addressed all other outstanding technical requirements, you may resubmit your manuscript within Editorial Manager.

We have replied to the email confirming that copyright permissions are not required and that all materials can be published under a Creative Commons license. Below, we provide our point-by-point reply.

Potential Copyright Issues:

i) Please confirm (a) that you are the photographer of 1A, and 1C, or (b) provide written permission from the photographer to publish the photo(s) under our CC BY 4.0 license.

We confirm that one of the authors (GM) is the photographer of 1A and 1C.

ii) Figures 1A, 1C, and 2A. Please confirm whether you drew the images / clip-art within the figure panels by hand. If you did not draw the images, please provide (a) a link to the source of the images or icons and their license / terms of use; or (b) written permission from the copyright holder to publish the images or icons under our CC BY 4.0 license. Alternatively, you may replace the images with open source alternatives. See these open source resources you may use to replace images / clip-art:
- <https://commons.wikimedia.org>

- <https://openclipart.org/>.

We drew some components by hand, while others were sourced from an open-access database (Openclipart, Creative Commons Zero 1.0 Public Domain License).

7) Your current Financial Disclosure states receiving many funds. However, your funding information on the submission form indicates only one fund. Please ensure that the funders and grant numbers match between the Financial Disclosure field and the Funding Information tab in your submission form. Note that the funders must be provided in the same order in both places as well.

We have ensured that the funders and grant numbers match between the Financial Disclosure field and the Funding Information tab in your submission form.

**Reviewers' comments**

**Reviewer #1:**

Thank you for the opportunity to review this manuscript. I really enjoyed reading it!
The study presents a well-structured and compelling investigation into multimodal integration in face-to-face communication, combining computational and experimental approaches. Specifically, the authors test whether Hierarchical Bayesian Causal Inference (HBCI) or Forced Fusion better explains human multimodal processing. I appreciate the authors' effort in using sophisticated methods to address such an important and complex question about the underlying mechanisms of multimodal communication. The topic is also both interesting and timely. However, I have some concerns related to the experimental design and stimuli, which I describe below.

We thank the reviewer for the positive assessment of the manuscript and the valuable feedback, which allowed us to elaborate on several aspects of our work. We believe that these additions have improved the clarity and transparency of the study, as discussed in more detail below.

1. The duration of communicative and non-communicative signals differs between experiments (1500ms vs. 500ms). The authors acknowledge that this likely led to higher auditory noise in the non-communicative condition in Exp. 1. While this was addressed in Exp. 2 by adjusting the duration of the non-communicative stimuli, it raises the question of why it was not controlled from the beginning. Although the authors mention their intention to use these materials in future neuroimaging studies, the duration difference remains a potential confound. Would it make sense to focus on Exp. 2 in the main text and summarize Exp. 1 in the SM instead? This is just a suggestion, and the authors may decide against it if they believe it does not improve the manuscript.

We appreciate the reviewer’s thoughtful suggestion regarding the structure of the manuscript. We agree that Experiment 2 addresses an outstanding point from Experiment 1 by equalizing the duration of the auditory stimuli and thus the associated sensory uncertainty. Crucially, by comparing the results of the two experiments, it is possible to realize that prior expectations interact with sensory uncertainty to determine the final perceptual estimates, in line with Bayesian Causal Inference principles. In Experiment 1, the ventriloquist effect increased in the communicative condition due to a stronger common cause prior. Concurrently, it increased in the non-communicative condition due to higher auditory uncertainty. When stimulus duration was equated across conditions in Experiment 2, removing differences in sensory uncertainty, we unveiled the direct influence of prior expectations on participants’ behaviour. For this reason, we believe that including both experiments in the main text strengthens the overall narrative of the manuscript. Moreover, another reviewer explicitly praised this structure, noting that a strength of the study is the two-experiment design, with the second experiment both addressing a shortcoming of the first and replicating its findings. Collectively, we believe that presenting both experiments in the main manuscript improves the clarity, rigor, and transparency of our work. Therefore, we prefer to keep this structure.

2. The study would benefit from additional discussion on the potential impact of stimulus properties, such as duration and the positioning of auditory and visual cues. Do we know whether—and if so, how—these angles influence processing and integration? While conducting new norming experiments may not be feasible at this stage, grounding these choices more explicitly in prior literature could strengthen the rationale.

We agree on the importance of grounding our design choices more explicitly in prior literature. The angles and spatial configurations used in our experiments mirror those employed in a previous study by Ferrari & Noppeney [1], which used a highly similar design and procedure. The study found that these spatial positions reliably influenced audiovisual integration, as quantified by the ventriloquist effect. We built directly on these findings to inform our stimulus design, leveraging a validated paradigm to ensure the robustness and interpretability of the results. We now explicitly refer to this prior work in the Methods section:

P. 6 Lines 172-176

*The auditory information (voice) and the visual bodily cues (video) were independently sampled from three positions along the azimuth (-9°, 0° or 9° of visual angles), generating 9 possible spatial combinations and 3 levels of spatial disparity (0°, 9°, 18° of visual angles; Fig 1b). These spatial configurations influence audiovisual integration reliably, as demonstrated by previous work using a highly similar paradigm [73].*

Similarly, the study assumes that shifting recorded videos (e.g., from right to left) effectively simulates (in)direct eye contact. However, subtle inconsistencies in gaze and speaker orientation might introduce unintended cues that affect multisensory integration. I am also not convinced that these manipulations are truly more naturalistic (e.g., how often does the speaker entirely avoid eye contact with their interlocutor in a natural conversation? When it does occur, it is often due to conversational interruptions or because the message is (re)directed at someone else). Providing further justification for this approach could help clarify its validity.

We thank the reviewer for raising this point. We realize that further clarification is needed regarding the nature and rationale of our stimulus design.

First, we would like to clarify that we did not simulate (in)direct eye contact by shifting recorded videos horizontally (e.g., from right to left). Instead, eye contact was manipulated via vertical head and gaze movements: in the communicative condition, the speaker starts with their head down and then raises it to establish direct gaze with the listener; in the non-communicative condition, the speaker keeps the head down. Second, the horizontal shifts of the speaker across three positions (−9°, 0°, +9°) were introduced to generate audiovisual spatial disparity between the location of the visual stimulus (video) and the location of the auditory stimulus (voice). This disparity is what drives the ventriloquist effect, which we used to quantify audiovisual integration.

To sum up, vertical shifts of head and gaze towards the listener contributed to signalling the speaker’s intention to communicate; horizontal displacements between the video and audio locations created spatial incongruency, thereby letting us measure the amount of ventriloquist effect. We then asked whether and how the ventriloquist effect was influenced by communicative (relative to non-communicative) audiovisual signals. Importantly, the axis of gaze movement (vertical) was orthogonal to the axis of audiovisual disparity (horizontal), thereby avoiding potential perceptual or attentional biases due to eye movements that may influence spatial localization along the horizontal plane.

Finally, we recorded the videos from three distinct camera angles depending on the horizontal position to ensure that the speaker’s head, gaze, and torso orientation always pointed to the position of the participant (Fig 1a). This was done to give the impression that the speaker was addressing the listener in the communicative condition from each of the three positions. In other words, whether the speaker appeared at −9°, 0°, or +9°, they addressed the listener directly, as is typically the case when starting a conversation in natural settings. This way, the participants would perceive the speaker’s movements as directed toward them.

We have now clarified these methodological details in the Methods section:

P. 5 Lines 143-150

*During the recording, the camera was placed in three different positions along the azimuth for the speaker to be on the right side, left side or at the centre of the screen, at respectively 9°, −9° and 0° of visual angle, to ensure that the speaker’s head, gaze, and torso orientation always pointed to the position of the participant (Fig 1a). This was done to give the impression that the speaker was addressing the listener in the communicative condition from each of the three positions. In other words, whether the speaker appeared at −9°, 0°, or +9°, they addressed the listener directly, as is typically the case when starting a conversation in natural settings. This way, the participants would perceive the speaker’s movements as directed toward them.*

P. 5 Lines 154-157

*To address the participant in the communicative condition, each video started with the speaker looking down with her head bowed, then she lifted her head vertically, looked at the camera and uttered the word. Conversely, in the non-communicative condition, the speaker kept the head down and then produced a meaningless vocalization [8].*

P. 6 Lines 186-192

*Vertical shifts of head and gaze towards the listener contributed to signalling the speaker’s intention to communicate; horizontal displacements between the video and audio locations created spatial incongruency, thereby letting us measure the amount of ventriloquist effect. We then asked whether and how the ventriloquist effect was influenced by communicative (relative to non-communicative) audiovisual signals. Importantly, the axis of gaze movement (vertical) was orthogonal to the axis of audiovisual disparity (horizontal), thereby avoiding potential perceptual or attentional biases due to eye movements that may influence spatial localization along the horizontal plane.*

3. I like the idea of including a word association task at the end of communicative blocks. However, it introduces another potential confound that could influence the results. For example, it is possible that semantic or pragmatic expectations are strengthening the common cause prior, rather than audiovisual integration alone. Would it have been beneficial (if possible at all) to include a comparable semantic task in the non-communicative condition to allow for a more direct comparison?

We thank the reviewer for this thoughtful observation. The central aim of our study was indeed to evaluate whether communicative (pragmatic) expectations contribute to strengthening the common cause prior. This would in turn increase the amount of audiovisual integration, as quantified by the ventriloquist effect. Our results support this hypothesis: we observed greater audiovisual integration in the communicative than in the non-communicative condition. In terms of the underlying computational mechanism, this effect was explained by an increase in the common cause prior.

In this context, we introduced the semantic task in the communicative condition to strengthen the impression that the speaker intended to engage in a communicative interaction by having something meaningful to communicate to the participant. While introducing a comparable semantic task in the non-communicative condition might be feasible and appealing as a control, it would risk undermining the very contrast the design was built to highlight between the two conditions. Even a neutral semantic task might cue participants to attribute some degrees of intentionality or communicative coherence to the non-communicative condition, introducing the risk of additional interpretative ambiguity. Given that this was, to our knowledge, the first study directly examining the impact of higher-order communicative expectations on audiovisual integration, we prioritised maximizing the conceptual contrast to establish a clear and solid proof-of-principle. We acknowledge, however, that this approach comes with potential trade-offs in terms of interpretative specificity. Accordingly, the Discussion section now highlights the utility of tightly controlled follow-ups to isolate the contribution of communicative intent from other factors. We believe this addition helps clarify the scope of the current findings and points to valuable avenues for future research:

P. 20 Lines 642-653

*Notably, in the present study, we created pragmatic correspondences by introducing relevant linguistic content and ostensive bodily signals (head and gaze movements). Our design intentionally maximized the contrast between communicative and non-communicative signals to establish a clear proof-of-concept for the influence of communicative intent on audiovisual causal inference. While this approach introduces both low- and high-level differences between conditions (such as stimulus dynamics, attentional demands, and semantic content), we view these as intrinsic features of real-world communicative contexts. In Experiment 2, we addressed one specific difference by equating stimulus durations across conditions, thereby reducing interpretive ambiguity. Future studies should incorporate more finely matched control conditions to better isolate the effects of communicative intent from other contributing factors such as attention, arousal, and stimulus complexity. Furthermore, it would be interesting to extend the current findings to other relevant crossmodal cues, such as semantic correspondences [30–34] and socio-emotional cues [91].*

4. I was wondering why the authors decided to use still frames and vowel vocalization in the non-communicative condition, rather than videos of non-communicative gestures with naturalistic vocalizations (e.g., yawning or coughing). Using such more naturalistic non-communicative stimuli might have led to a more balanced comparison.

Following from the previous point, our decision to maximize the contrast between the communicative and non-communicative conditions was intended to establish a clear proof-of-concept. In this context, we decided against a control condition with dynamic videos (e.g. yawning or coughing with naturalistic vocalization) because previous work has shown that even putatively non-communicative gestures can be contextually interpreted as social signals (e.g. express boredom or insecurity) [2]. However, we acknowledge that this approach limits the specificity of our conclusions regarding the underlying mechanisms. Accordingly, we have now emphasized the need for future work using more tightly controlled conditions to isolate the contribution of communicative intent from other non-specific factors:

P. 22 Lines 650-651

*Future studies should incorporate more finely matched control conditions to better isolate the effects of communicative intent from other contributing factors such as attention, arousal, and stimulus complexity.*

5. From the figures, it appears that when an auditory stimulus was heard from the right, the headphones were displayed on the right, and similarly, when the speaker looked from the right, her video was also positioned on the right (and similarly for the left side and middle locations). Could the authors clarify the rationale for this design choice? An alternative approach might have been to present a central video with audio playing in the background. If the stimuli were always positioned congruently, participants might not need to truly integrate the signals but rather just recall their spatial locations. If I have misunderstood the trial structure, I apologize—please consider clarifying this in the figure or text to ensure other readers are not similarly confused.

We appreciate the reviewer’s comment and recognize that the rationale and structure of our stimulus design could benefit from additional clarification.

The auditory information (voice) and the visual bodily cues (video) were independently sampled from three positions along the azimuth (−9°, 0°, +9°), generating 9 possible spatial arrangements as represented in Fig 1b. In other words, we presented all possible combinations of auditory and visual positions along the horizontal plane. In three of them, the auditory and visual stimuli were spatially congruent, i.e. sampled from the same spatial position (Fig 1b, white cells). In the remaining combinations, the auditory and visual stimuli were spatially incongruent, i.e. sampled from different spatial positions (Fig 1b, light and dark grey cells).

Importantly, we decided to introduce horizontal shifts between the video location and the audio location (instead of, for instance, a central video with audio playing in the background) because this spatial configuration has been extensively validated in similar past research (see e.g. [1]). We built directly on this prior literature to ensure the robustness and interpretability of the results. Following a previous comment from the reviewer, we now explicitly refer to this:

P. 6 Lines 172-176

*The auditory information (voice) and the visual bodily cues (video) were independently sampled from three positions along the azimuth (-9°, 0° or 9° of visual angles), generating 9 possible spatial combinations and 3 levels of spatial disparity (0°, 9°, 18° of visual angles; Fig 1b). These spatial configurations influence audiovisual integration reliably, as demonstrated by previous work using a highly similar paradigm [73].*

To visualize the experimental procedure, Fig 1c exemplifies a few representative audiovisual spatial combinations from all those employed in our study. We would like to clarify that the headphones icon was not actually presented on the screen during the experiments, but simply represents the audio location for visualization purposes in the figure. In other words, for each trial, participants saw the visual component on the screen and heard the voice in their headphones. We have now clarified all the above aspects in the figure caption:

Fig 1 Caption

*b) We used a 3 (visual stimuli position) × 3 (auditory stimuli position) × 2 (action intention) × 2 (response modality) factorial design, resulting in 36 total experimental conditions. We presented all 9 possible combinations of auditory and visual positions, generating 3 levels of spatial disparity: spatially congruent, i.e. sampled from the same spatial position (0°, white cells); low AV disparity (9°, light grey cells); high AV disparity (18°, dark grey cells). c) The trial started with a 500 ms fixation cross at the centre of the screen, followed by the synchronous audiovisual stimulus. In communicative blocks, the stimuli were videos of the speaker addressing the participant and uttering a word, which lasted on average 1500 ms (range 1000-2000 ms) depending on word length. In non-communicative blocks, the stimuli lasted 500 ms in Experiment 1 and 1500 ms in Experiment 2. After the stimuli offset, a letter on the screen (‘A’ for auditory and ‘V’ for visual) cued participants to report the auditory or visual position using a specific keyboard key (1500 ms response window). At the end of communicative blocks, participants were prompted by the letter ‘P’ on the screen (for ‘parola’, i.e. ‘word’ in Italian) to say a word representing the general theme that connected the words in the block. The experiment consisted of 4 runs, each containing 18 blocks (9 communicative and 9 non-communicative) and 9 trials per block. Please note that the figure exemplifies a few representative audiovisual spatial combinations from all those employed in the study. For each trial, participants saw the visual component on the screen and heard the voice in their headphones. The headphones icon represents the audio location for visualization purposes (it was not presented on the screen during the experiments).*

I also have some additional discussion points that the authors may wish to consider.

6. The authors argue that enhanced attention due to communicative salience could still be accounted for by Forced Fusion, as increased reliability strengthens integration. While the study suggests that HBCI uniquely explains the findings, was an alternative Forced Fusion model with an attention-weighted reliability component explicitly ruled out? Addressing this could further solidify the conclusions.

We thank the reviewer for raising this crucial point. Our model space does indeed include an alternative to the HBCI model that specifically addresses the alternative computational mechanism highlighted by the reviewer. In particular, we implemented a “FF Separated model” with two separate visual and auditory reliability parameters for each action intention condition (communicative: σ_V_ Com and σ_A_ Com; non-communicative: σ_V_ NCom and σ_A_ NCom). Thanks to this architecture, the model would account for a purely attention-driven change in sensory reliability due to communicative salience. This model was directly compared to HBCI to characterize the exact computational mechanism by which communicative signals may increase multisensory integration. On the one hand, the perceptual characteristics of the audiovisual stimuli may influence the reliability of the sensory information before integration in both the FF and the BCI models. Specifically, the speaker may capture the observer's attention more effectively by addressing them with head and gaze (communicative condition) as opposed to keeping the head down (non-communicative condition), thereby decreasing the noise (i.e. increasing the reliability) of the attended visual information. This would be captured by a decrease of σ_V_ in the communicative than non-communicative condition, which would increase the visual weight during the fusion process under the communicative condition. Alternatively, the shared communicative nature of the video and audio stimuli may reinforce the prior belief that the two signals come from a common cause, as captured by an increase of the parameter p_common_ in the BCI model for communicative than non-communicative trials. Across the two experiments, we showed that the BCI model provided a significantly better fit to the data. Further, Experiment 2 confirmed that a modulation in prior expectations explained our findings, instead of a purely attention-driven change in sensory reliability. We presented our modelling rationale in the Methods section:

P. 11 Lines 366-384

*By comparing FF vs BCI models, we evaluated whether observers always integrate auditory and visual stimuli regardless of causal uncertainty (in line with FF), or if they combine the two alternative perceptual estimates weighted by the posterior probabilities of the respective causal structure (in line with BCI) as described by Eq. 6 and 7. This would confirm that prior expectations arbitrate between the integration and segregation of vocal and bodily signals during face-to-face communication [4,5]. By comparing Pooled vs Separated models, we evaluated whether observers showed a stronger tendency to integrate vocal and bodily information for communicative than non-communicative signals. Crucially, by comparing the BCI versus FF Separated models we characterized the exact computational mechanism by which communicative signals may increase multisensory integration. On the one hand, the perceptual characteristics of the audiovisual stimuli may influence the reliabilities of the sensory information before integration in both the FF and the BCI models. Specifically, the speaker may capture the observer's attention more effectively by addressing them with head and gaze (communicative condition) as opposed to keeping the head down (non-communicative condition), thereby decreasing the noise (i.e. increase the reliability) of the attended visual information [73]. This would be captured by a decrease of 𝜎_𝑉_ in the communicative than non-communicative condition, which would increase the visual weight during the fusion process under the communicative condition. Alternatively, the shared communicative nature of the video and audio stimuli may reinforce the prior belief that the two signals come from a common cause, as captured by an increase of p_common_ in the BCI model for communicative than non-communicative trials. The factorial model comparison allowed us to directly arbitrate between these competing hypotheses.*

In line with the reviewer’s suggestion, we contextualize our findings in light of our modelling rationale in the Discussion section:

P. 19 Lines 619-628

*We contrasted two plausible accounts for how we parse the conversational scene into coherent multisensory units while segregating concurrent, yet unrelated, signals. On the one hand, observers may arbitrate between integration and segregation by taking into account the uncertainty about the underlying causal structure, as predicted by Bayesian Causal Inference [18,52–56]. In this framework, prior expectations would mediate whether sensory inputs are integrated into a unified percept or treated independently. On the other hand, observers may follow Forced Fusion principles [60–62], mandatorily integrating audiovisual signals regardless of causal uncertainty. Here, communicative bodily movements may attract greater attention than non-communicative ones due to higher perceptual salience [64–70] or social relevance [71,72]. Since attention decreases sensory uncertainty and thereby increases the weight of attended signals during the fusion process [73], communicative visual cues may guide the parsing of the conversational scene.*

7. A more conceptual question—if non-communicative signals (or noise) are not part of the conversation (i.e., they are not integrated) in the first place, is there a strong justification for modeling them within a multisensory integration framework?

We thank the reviewer for this insightful conceptual question, which gives us the opportunity to further clarify the aim of the study. Non-communicative crossmodal signals may still be subject to multisensory integration if they appear close to each other in time or space, as extensively documented in previous studies (e.g. [1,3]) using non-communicative artificial stimuli (e.g. beeps and flashes). In other words, non-communicative crossmodal correspondences (e.g. temporal synchrony and spatial congruency) are known to drive crossmodal binding and influence perceptual inference. Thus, non-communicative stimuli provide a meaningful baseline within a multisensory integration framework. The core question we aimed to address is whether the addition of communicative intent strengthens this tendency toward integration, compared to when the signals are non-communicative but closely matched in timing and spatial configuration. We have clarified this rationale in the revised Introduction:

P. 4 Lines 114-121

*We presented participants with communicative and non-communicative audiovisual stimuli and quantified audiovisual integration through the spatial ventriloquist effect [74]. Since non-communicative crossmodal correspondences (e.g. spatial congruency) are known to drive crossmodal binding and influence perceptual inference [27-29], non-communicative stimuli provided a meaningful baseline within a multisensory integration framework. The core question we aimed to address is whether the addition of communicative intent strengthens the tendency toward integration, compared to when the signals are non-communicative but closely matched in timing and spatial configuration.*

Finally, I have a minor suggestion, if the journal guidelines allow and the authors agree: a table summarizing key terminology (e.g., ventriloquist effect, HBCI, Forced Fusion, etc.) might be a helpful addition for readers less familiar with the field.

We appreciate the reviewer’s suggestion. While the journal guidelines do not mention the possibility of terminology tables, we have ensured that all key terms are clearly introduced and defined in the text. Additionally, we have added detailed legends to all figures and tables to enhance clarity and support readers who are less familiar with the field.

**Reviewer #3:**

In two psychophysical experiments, the study investigates whether near-natural audiovisual communication signals are integrated or segregating depending on their communicative intent. Using computational modelling, the authors report that integration/segregation follows the principles of Bayesian causal inference (BCI), which has been so far mostly applied to simplified non-social audiovisual signals. More specifically, the author find that commucitive signals (compared to a non-communicative control conditions) increase the BCI model’s causal prior parameter.
Overall, the study investigates a very interesting and urgent research question (causal inference on social AV signals), applies well implemented experimental stimuli and design, and thoroughly models and analyses behavioral data. The manuscript is very well written and nice to read. A further strength is that the authors performed two experiments, the second to address a shortcoming of the first experiment (which was unequal duration of A stimuli between communication conditions) and to replicate results from the first experiment. My major concern is the implementation of the control condition: While the experimental condition with communicative signals uses a video with meaningful speech signals and semantic secondary task, the non-communicative control condition only used static pictures with vowel sounds. This leaves both conditions unmatched in many aspects beyond ‘communicative’ signals (stimulus dynamics, differential secondary task etc.). This introduces many potential confounds (e.g. attention/salience) which are known to also influence audiovisual integration and specifically the causal prior.

We sincerely thank the reviewer for their thoughtful and constructive feedback, as well as for the positive appraisal of our manuscript. We particularly appreciate the recognition of the study’s conceptual contribution, methodological rigor, and clarity of presentation. The reviewer’s comments have been invaluable in helping us refine the manuscript, and we believe the revisions undertaken in response have substantially enhanced the clarity, transparency, and interpretability of our findings, as detailed below.

My more specific comments:

Methods:
• Auditory spatial stimuli: The authors used HRTF to create virtual spatial sounds. Which head model did they use?

We have added this important information in the Methods section:

P. 6 Lines 162-165

*The auditory stimuli were convolved with spatially selective head-related transfer functions (HRTFs) derived from the ARI HRTF Database [76]. The database contains high-resolution measurements across over 250 subjects for 1,550 spatial positions, from which we selected the ones relevant to the study.*

How well were the A spatial stimuli localizable?

In Experiment 2, we introduced a unisensory auditory screening before the main task to ensure participants could distinguish and correctly localize the auditory stimuli without visual interference. We recruited 41 volunteers, 7 of which were excluded (i.e. they did not perform the main task) due to poor auditory localization. We explain these details in the Methods section:

P. 16 Lines 529-538

*We included a unisensory auditory screening to ensure participants could distinguish and correctly localize the auditory stimuli without visual interference. This way, any effects in the main experiment could be more safely interpreted in terms of visual capture of perceived sound location, instead of a response that arises from perceptual confusion or uncertainty. Observers located auditory signals – the same auditory stimuli presented during the experiment – randomly presented at −9˚, 0˚, or 9˚ visual angle along the azimuth. Their auditory localization accuracy was quantified by the root mean square error (RMSE) between the participants’ reported location and the signal’s true location (low RMSE indicates high accuracy). Based on similar research [73], observers were excluded as outliers if their RMSE was greater than 6˚. We recruited 41 volunteers, 7 of which were excluded due to poor auditory localization.*

For the 34 participants included in the study (our target sample), auditory localization was reliable, as visualized below (proportion correct responses = 0.81 ± 0.02, mean ± SEM; Pearson’s correlation coefficient between true and reported location: r = 0.89 [0.86, 0.92], mean ± 95% CI). We have added these details to the manuscript:

P. 16 Lines 538-541

*As a result, the final analyses included 34 participants (26 females; mean age 23, range 18-47 years), who demonstrated reliable auditory localization (proportion correct responses = 0.81 ± 0.02, mean ± SEM; Pearson’s correlation coefficient between true and reported location: r = 0.89 [0.86, 0.92], mean ± 95% CI).*

• Control condition of static picture with a single auditory vowel and without secondary task: This choice introduces many low level (e.g. visual motion) and higher-level (e.g. attention/saliency, dual task demands, arousal etc.) differences compared to the dynamic video in the experimental condition. Further, the non-communicative pictures are much shorter than videos (500 ms vs. 1-2 s), which was corrected in Exp. 2. Because communicate videos even conveyed a short “story” over trials in a block, there is even a semantic difference. While this is a creative idea to increase participants’ motivation, it also makes the control condition even less comparable. Overall, this choice introduces many low- and high-level confounds, which are partially already known to influence AV integration and also BCI model parameters. For example, attention affects the causal prior (Badde 2020) and variance parameters (their own study, Ferrari & Noppeney 2021), so that differential attentional processes may also explain the results. So I think a more comparable control conditions would have allowed to more specific conclusions whether really the social signals make the difference. This may be even more important for a future neuroimaging study (which seems to be planned).

We thank the reviewer for this important comment. We fully acknowledge that our control condition differs from the experimental condition along several dimensions—including stimulus dynamics, attentional demands, and semantic richness—which may introduce both low- and high-level confounds.

Our design was intentionally structured to maximize the contrast between communicative and non-communicative signals, thereby establishing a clear proof-of-concept for the influence of communicative intent on audiovisual causal inference. Rather than treating this as a limitation per se, we see it as a theoretically motivated choice: the use of static images paired with a meaningless vocalization, devoid of narrative context or interactional cues, created a maximally “non-communicative” baseline. This enabled us to observe whether and how communicative context can *tip* the BCI model’s causal prior in a robust and detectable way.

Importantly, the dual-task design—requiring participants to extract semantic content from the communicative stimuli—was not simply an added cognitive burden, but a deliberate attempt to simulate real-world communicative interaction. We agree that this introduces attentional and cognitive differences compared to the control condition. However, we would argue that these are not merely confounds, but integral aspects of what makes communication *communicative*. In that sense, the additional demands placed on participants in the experimental condition reflect naturalistic features of social perception that are often overlooked in traditional multisensory integration paradigms.

We also note that Experiment 2 was designed to address one of the specific concerns raised by the reviewer—the unequal stimulus duration—by equating presentation times across conditions. While this does not eliminate all differences, it helps narrow the interpretive gap.

Nonetheless, we agree that future work should adopt more finely matched control conditions to disentangle communicative intent from attentional load, stimulus dynamics, and arousal. We now highlight this explicitly in the revised Discussion section as a key direction for future research:

P. 19 Lines 642-653

*Notably, in the present study, we created pragmatic correspondences by introducing relevant linguistic content and ostensive bodily signals (head and gaze movements). Our design intentionally maximized the contrast between communicative and non-communicative signals to establish a clear proof-of-concept for the influence of communicative intent on audiovisual causal inference. While this approach introduces both low- and high-level differences between conditions (such as stimulus dynamics, attentional demands, and semantic content), we view these as intrinsic features of real-world communicative contexts. In Experiment 2, we addressed one specific difference by equating stimulus durations across conditions, thereby reducing interpretive ambiguity. Future studies should incorporate more finely matched control conditions to better isolate the effects of communicative intent from other contributing factors such as attention, arousal, and stimulus complexity. Furthermore, it would be interesting to extend the current findings to other relevant crossmodal cues, such as semantic correspondences [30–34] and socio-emotional cues [91].*

• Audiovisual weight index:

o Why was the wAV normalized by the average reported location in audiovisual congruent trials computed across participants? The normalization helps to account for individual response biases (e.g. a central tendency), but when computing it with the across-participants average, individual response biases are not accounted for. I would suggest to make a sensitivity analyses whether this choice (individual vs. group-mean response location) affects the results.

We agree with the reviewer that individual response biases are not accounted for when computing the normalization using the reported location in audiovisual congruent trials averaged across participants. Additionally, we averaged across the four (action intention × response modality) experimental conditions. To account for individual response biases in the most stringent way, we have now computed the w_AV_ using the participant-specific and condition-specific average reported location in audiovisual congruent trials. Results remained consistent with the previous version, strengthening the robustness of the effects. We have added this sensitivity analysis to the supplementary materials (S3 Fig) and referenced it in the Results section of the main text:

P. 14 Lines 446-450

*Importantly, response biases may vary across participants and experimental conditions. To account for these individual biases more stringently, we recomputed the w_AV_ using the average reported location in audiovisual congruent trials for each participant and each of the four (action intention × response modality) experimental conditions. This sensitivity analysis (S3 Fig) yielded results consistent with the original analysis, confirming the robustness of our findings.*

P. 18 Lines 584-586

*Moreover, a sensitivity analysis using the average reported location in audiovisual congruent trials for each participant and each of the four (action intention × response modality) experimental conditions confirmed the robustness of this effect (S3 Fig).*

o wAV is analysed with non-parametric permutation tests, but important details how this permutation test was implemented is missing (e.g. permutation scheme for main/interaction effects, test statistic used etc.)

Thank you for pointing out this inaccuracy. We have added this important information to the Methods section:

P. 9 Lines 275-283

*To avoid making parametric assumptions, we evaluated the main effects of response modality, spatial disparity, action intention and their interactions using non-parametric permutation tests at the between-subject level. The observed test statistic was the across-participants’ difference in means between conditions (or condition combinations for interaction effects). To construct the null distribution, we randomly permuted the condition labels across participants, preserving the number of participants per condition, and recalculated the test statistic for each permutation. This process was repeated 4,096 times to generate an empirical null distribution. Two-tailed p-values were computed by comparing the observed test statistic to the null distribution, calculating the proportion of permuted statistics that exceeded the observed value.*

• BCI modelling: The authors only compare the BCI model to a forced fusion model, which is a rather weak model when spatial disparities are introduced (because the model’s common cause assumption is obviously wrong). Stronger / equally complex models are stochastic fusion and fixed criterion model (Acerbi et al. 2018 Plos Comp Biol), the latter can also accommodate spatial disparity in a (non-optimal) fashion. Thus, the authors could expand their model space. An additional interesting idea to explore with more model candidates would be that participants may also switch their perceptual decision strategy (e.g. from causal inference to stochastic fusion) depending on the communicative signals.

We are grateful to the reviewer for suggesting the inclusion of the stochastic fusion model and the fixed criterion model [4]. We have carefully considered these additional model candidates and fully acknowledge that they offer psychologically plausible heuristic approximations of multisensory decision-making, particularly in accommodating the effect of spatial disparity. We also agree that models capturing decision strategies of different complexity may also provide valuable insights in contexts where strategy use could vary with stimulus properties or task demands. However, the central goal of our study was to test whether and how communicative intent modulates causal inference depending not only on spatial disparity but also on modality-specific sensory uncertainty. To this end, we deliberately employed a modelling framework grounded in normative Bayesian inference because of its sensitivity to sensory and causal uncertainty, which was critical in our design for two key reasons.

First, we sought to contrast two competing hypotheses that require sensitivity to sensory and causal uncertainty. On the one hand, observers may arbitrate between integration and segregation by taking into account the uncertainty about the underlying causal structure, as predicted by BCI. In this framework, prior expectations would mediate whether sensory inputs are integrated into a unified percept or treated independently. On the other hand, observers may follow forced fusion principles, mandatorily integrating audiovisual signals weighted by their relative sensory reliabilities but regardless of causal uncertainty. Here, communicative bodily movements may attract greater attention than non-communicative ones due to higher perceptual salience [5–11] or social relevance [12,13]. Since modality-specific attention decreases sensory uncertainty and thereby increases the weight of attended signals during the fusion process [1], communicative visual cues may guide the parsing of the conversational scene.

Second, sensitivity to sensory and causal uncertainty was a key feature in our design in light of the different stimulus durations in Experiment 1. By comparing the results of the two Experiments, it is possible to realize that prior expectations interacted with sensory uncertainty to determine the final perceptual estimates, in line with BCI. In Experiment 1, the ventriloquist effect increased in the communicative condition due to a stronger common cause prior. Concurrently, it increased in the non-communicative condition due to higher auditory uncertainty. When stimulus duration was equated across conditions in Experiment 2, removing differences in sensory uncertainty, we unveiled the direct influence of prior expectations on participants’ behaviour.

Below, we clarify why the stochastic fusion and fixed criterion models, while compelling in their own right, are less suited for addressing our core hypotheses within our experimental paradigm.

The stochastic fusion model exhibits lack of sensitivity to spatial disparity, similarly to the forced fusion model. However, contrary to forced fusion, stochastic fusion does not mandatorily perform integration. On each trial, it makes a random choice: either it fuses the signals or it segregates them. Because it sometimes performs segregation, stochastic fusion can partially accommodate scenarios where observers do not always integrate. Critically, however, the integration decision itself is random, not based on spatial disparity or sensory uncertainty [4].

The fixed criterion model exhibits sensitivity to spatial disparity by applying a static disparity threshold to arbitrate between integration and segregation, disregarding the relative sensory uncertainties. This is particularly maladaptive in tasks where modality-specific variances differ substantially [14]. If the auditory cue is highly uncertain (as is the case of spatial localisation), a large audiovisual disparity might still plausibly arise from a common source, but the fixed criterion model would nonetheless reject it. By contrast, the optimal BCI decision function (model averaging) integrates the estimates of each causal structure weighted by the posterior probability of the respective structure, considering the relative sensory uncertainties. If spatial disparity is explainable by noise, BCI may still infer a common cause (see Simulation Figure below).

Collectively, while the stochastic fusion model and the fixed criterion model are not ideally suited to the aims and characteristics of the present study, the normative BCI and forced fusion strategies offer the most appropriate and interpretable framework. We agree, however, that expanding the model space in future studies (e.g. in experimental contexts that better match sensory reliability across the senses) would offer a crucial direction for further investigation. We have included this important aspect in the revised Discussion:

P. 20 Lines 661-672

*Note that we deliberately employed a modelling framework grounded in normative Bayesian inference because of its sensitivity to both sensory and causal uncertainty. This was a key feature of our model space given the substantial mismatch in sensory reliability between the auditory and visual modalities. Within this framework, the forced fusion model served as a baseline comparison but represented a relatively weak alternative to BCI: given the presence of varying spatial disparities, the model’s common-cause assumption was unlikely to hold. Alternative models (e.g. stochastic fusion, fixed criterion [55]) offer psychologically plausible heuristic approximations of multisensory perceptual inference that can better accommodate the effect of spatial disparity, albeit compromising sensitivity to both sensory and causal uncertainty. Models capturing decision strategies of different complexity may also provide valuable insights in contexts where strategy use could vary with stimulus properties or task demands. Expanding the model space to include such alternatives is therefore an important direction for future research.*

**Simulation Figure**: Predicted auditory location estimate ($\hat{S_{A}}$) as a function of absolute audiovisual disparity derived from the BCI model (blu dots) and the fixed criterion model (red dots). For better visualization, we plot the continuous estimates before response discretization. The horizontal lines represent the 3 possible response locations in our experiment. The auditory and visual inputs were sampled from independent Gaussian distributions (10,000 simulations; V: μ_V_ = 9°, σ_V_ = 2°; A: μ_A_ = 0°, σ_A_ = 8°). The fixed criterion threshold was set at 6° as a plausible representative value. A = auditory; V = visual.

• The non-parametric comparison of model parameters could be implemented with a permutation test, which should provide better power compared to a Wilcoxon signed-rank test.

• If possible, the authors may consider reporting Bayes factors where applicable to provided also evidence for null effects (e.g. the parameter comparison)

We agree with the reviewer’s suggestion and have replaced Wilcoxon signed-rank tests with between-subject permutation tests. Additionally, we have supplemented these analyses with Bayes factors to provide further evidence for the interpretation of the results. The corresponding sections in the Methods and Results have been updated accordingly:

P. 13 Lines 422-426

*To refrain from parametric assumptions, we employed non-parametric permutation tests at the between-subject level (same statistical procedure as for the w_AV_ index). For effect sizes, we report the difference between the across-participants’ mean empirical effect and the mean of the nonparametric null distribution and 95% CI [73]. Finally, we supplement these analyses with Bayes factors (BF_10_) from Bayesian non-parametric pairwise tests implemented in JASP 0.19.1.*

P. 15 Lines 480-491

*Results showed that the common cause prior p_common_ was significantly higher in the communicative relative to non-communicative condition (p = 0.003, es = 0.08, CI(95%) = [0.02, 0.15], BF_10_ = 155.6) suggesting that observers held a greater a priori expectation that audiovisual signals originate from a common cause in a communicative context. Instead, the visual noise 𝜎_𝑉_ did not differ across the two action intention conditions (p = 0.466, es = 0.18, CI(95%) = [-0.09, 0.44], BF_10_ = 0.49). Hence, the perceptual characteristics of the visual stimuli (communicative: dynamic video of speaker addressing the observer; non-communicative: static frame of speaker looking down) did not modulate visual reliability and thus did not change the weight assigned to the visual modality during audiovisual integration across the two conditions. Critically, however, the auditory noise 𝜎_𝐴_ was significantly higher in the non-communicative condition (p = 0.047, es = -0.85, CI(95%) = [-1.61, -0.10], BF_10_ = 10.3), indicating that the auditory information was more noisy (i.e. less reliable) and thereby carried less weight during the fusion process (for comprehensive results, see S2 Table).*

P. 18 Lines 591-596

*Further, we also confirmed that the common cause prior p_common_ increased in the communicative relative to the non-communicative condition (p = 0.001, es = 0.11, CI(95%) = [0.04, 0.18], BF_10_ = 259.6) (Fig 3c). Conversely, we found no significant difference between the two action intention conditions for the auditory noise 𝜎_𝐴_ (p = 0.213, es = 0.47, CI(95%) = [-0.36, 1.29], BF_10_ = 0.58) and visual noise 𝜎_𝑉_ (p = 0.174, es = -0.46, CI(95%) = [-0.89, -0.03], BF_10_ = 0.85) parameters (for comprehensive results, see S2 Table).*

• I highly appreciate that the authors share documented code and data (good open science practices!).

Thank you for the very positive feedback.

Results:
• Exp. 1: I find it surprising that the communicative condition does not modulate wAV, but it strongly affects the causal prior and the auditory variance model parameters. Why do we not see this in the wAV index? E.g. a higher causal prior in the communicative condition should translate into a higher wAV for A report. This inconsistency deserves further exploration… I think the author should also plot model predictions for wAV to understand this inconsistency. It is even more striking because higher causal prior (= more V bias) and lower auditory variance (= more V bias) for communicative signals influence wAV in the same direction for A report, towards stronger V influence (as found in Exp. 2). So, I agree with the author’s BCI model interpretation that a larger causal prior increases multisensory integration for both A and V report, but I disagree with the authors’ interpretation that larger auditory variance increases multisensory integration in general: larger auditory variance generally increases the visual / decreases the auditory influence for both A and V report (i.e. a vertical shift in wAV for both tasks). For A report, this increased wAV can be interpreted as stronger integration, but for V report, the (anyhow tiny) A influence is even further reduced, which means stronger segreation (theoretically within the BCI model). Thus, shifts in A or V variance do not generally increase / decrease multisensory integration.

We thank the reviewer for this insightful comment, and we acknowledge that our initial wording was inaccurate. We did not mean to suggest a general increase in integration across both report modalities. On the contrary, in line with the reviewer’s comment, we intended to convey that increased auditory noise enhances multisensory integration specifically for auditory report.

We would like to clarify a further point regarding Experiment 1. We believe that we did not find an effect of action intention on the w_AV_ under auditory report because two opposing forces were influencing the final localization response. On the one hand, multisensory integration was enhanced in the communicative condition through a stronger common cause prior. On the other hand, the visual capture of sound was enhanced in the non-communicative condition due to higher auditory noise. A decrease in auditory reliability decreased the weight of the auditory information and thereby its influence on the perceived sound position, thereby determining a stronger visual capture. In other words, we believe that, for auditory report, higher causal prior (= more V bias) and lower auditory variance (= more A bias) for communicative signals influenced the w_AV_ in opposite directions and thereby cancelled any potential effects on the w_AV_, in line with the principles of BCI. This conclusion is further corroborated by the good fit between the empirical and predicted w_AV_ values, as now shown in Supplementary S3 Fig (we thank the reviewer for this thoughtful suggestion).

In Experiment 2, we eliminated the auditory noise difference across the two action intention conditions by matching the stimulus duration. In other words, we no longer had lower auditory variance (= more A bias) for communicative signals. This revealed that a higher causal prior for communicative than non-communicative signals increased the visual capture of perceived sound position (= more V bias).

We have restructured the Results section of both experiments to incorporate these important clarifications:

P. 15 Lines 497-503

*Together, it is then plausible that two opposing forces across the two action intention conditions interacted to shape the spatial perceptual estimates, ultimately neutralizing any effects on the final response (and, thus, on the w_AV_ index). On the one hand, multisensory integration was enhanced in the communicative condition through a stronger common cause prior. On the other hand, the visual capture of sound was enhanced in the non-communicative condition due to higher auditory noise. A decrease in auditory reliability decreased the weight of the auditory information and thereby its influence on the perceived sound position, thereby determining a stronger visual capture.*

P. 18 Lines 601-604

*Together, these findings corroborate the hypothesis that two contrasting forces were at play in Experiment 1. On the one hand, multisensory integration was enhanced in the communicative condition through a higher common cause prior. On the other hand, the visual capture of sound was enhanced in the non-communicative condition due to higher auditory noise.*

• Exp. 2: It is good to see that now wAV and parameter comparison align, when the duration of the A stimulus in both communication conditions is matched. One way to test this ‘parameter – wAV ’ link more formally would be to correlate with individual causal priors / auditory variances with the individual communication signal effect on wAV (also in Exp. 1).

In agreement with the reviewer’s suggestion, we have performed a follow-up correlation analysis to test the functional connection between model parameters and w_AV_. For both Experiments, we confirm that individual increases of w_AV_ for the communicative relative to the non-communicative condition (w_AV_ Com > w_AV_ NCom) *positively* correlate with the respective effect on the causal prior (p_common_ Com > p_common_ NCom) and auditory noise (𝜎_𝐴_ Com > 𝜎_𝐴_ NCom). These findings nicely dovetail with the rationale outlined above for Experiment 1. For auditory report, *higher* causal prior (= more V bias) and *lower* auditory variance (= more A bias) for communicative signals influenced the w_AV_ in opposite directions and thereby cancelled any potential effects on the w_AV_, in line with the principles of BCI. We have now expanded the Results section to include the correlation analysis (see also Fig 4):

P. 15 Lines 492-503

*To formally test the functional connection between the model parameters and the w_AV_, we performed a follow-up correlation analysis (Fig 4). In line with the principles of BCI, we found that individual increases of w_AV_ for the communicative relative to the non-communicative condition (w_AV_ Com > w_AV_ NCom) positively correlated with the respective effect on the causal prior (p_common_ Com > p_common_ NCom) and auditory noise (𝜎_𝐴_ Com > 𝜎_𝐴_ NCom). Both correlations were highly significant (p_common_: r = 0.78, p < 0.001; 𝜎_𝐴_: r = 0.69, p < 0.001). Together, it is then plausible that two opposing forces across the two action intention conditions interacted to shape the spatial perceptual estimates, ultimately neutralizing any effects on the final response (and, thus, on the w_AV_ index). On the one hand, multisensory integration was enhanced in the communicative condition through a stronger common cause prior. On the other hand, the visual capture of sound was enhanced in the non-communicative condition due to higher auditory noise. A decrease in auditory reliability decreased the weight of the auditory information and thereby its influence on the perceived sound position, thereby determining a stronger visual capture.*

P. 18 Lines 596-604

*Again, we performed a follow-up correlation analysis between the model parameters and the w_AV_ (Fig 4). Following the principles of BCI, we found that individual increases of w_AV_ for the communicative relative to the non-communicative condition (w_AV_ Com > w_AV_ NCom) positively correlated with the respective effect on the causal prior (p_common_ Com > p_common_ NCom) and auditory noise (𝜎_𝐴_ Com > 𝜎_𝐴_ NCom). Both correlations were highly significant (p_common_: r = 0.79, p < 0.001; 𝜎_𝐴_: r = 0.50, p = 0.002). Together, these findings corroborate the hypothesis that two contrasting forces were at play in Experiment 1. On the one hand, multisensory integration was enhanced in the communicative condition through a higher common cause prior. On the other hand, the visual capture of sound was enhanced in the non-communicative condition due to higher auditory noise.*

As above, I would not state that higher auditory noise enhances multisensory integration in general (only for A report this makes sense because the stronger V bias is a signature of MI).

Following from above, we acknowledge that our initial wording was inaccurate. We did not mean to suggest a general increase in integration across both report modalities. On the contrary, we agree that increased auditory noise should enhance multisensory integration specifically for auditory report. We have revised the manuscript to clarify this point:

P. 15 Lines 499-503

*On the one hand, multisensory integration was enhanced in the communicative condition through a stronger common cause prior. On the other hand, the visual capture of sound was enhanced in the non-communicative condition due to higher auditory noise. A decrease in auditory reliability decreased the weight of the auditory information and thereby its influence on the perceived sound position, thereby determining a stronger visual capture.*

P. 18 Lines 602-604

*On the one hand, multisensory integration was enhanced in the communicative condition through a higher common cause prior. On the other hand, the visual capture of sound was enhanced in the non-communicative condition due to higher auditory noise.*

Discussion:
• I think the authors need to acknowledge the limitation that the communicative videos could increase the common cause prior due to confounds/unspecific mechanisms, such as attention/saliency/arousal/dual task demands etc.. The authors state that they wanted to maximize the difference between the communicative and non-communicative conditions, but I think this decision comes at the price of many potential confounds, quite strongly limiting the specificity of their conclusions.

As discussed earlier, we fully agree that the two action intention conditions (communicative vs. non-communicative) vary across multiple dimensions concurrently, such as saliency, arousal/attention and dual task setting. Our decision to maximize the contrast between the communicative and non-communicative conditions was intended to establish a clear proof-of-concept, but we acknowledge that this approach introduces potential confounds that limit the specificity of our conclusions regarding the underlying mechanisms. We have now added a clearer discussion of these limitations in the revised manuscript and emphasized the need for future work using more tightly controlled conditions to isolate the contribution of communicative intent from other non-specific factors.

P. 19 Lines 642-653

*Notably, in the present study, we created pragmatic correspondences by introducing relevant linguistic content and ostensive bodily signals (head and gaze movements). Our design intentionally maximized the contrast between communicative and non-communicative signals to establish a clear proof-of-concept for the influence of communicative intent on audiovisual causal inference. While this approach introduces both low- and high-level differences between conditions (such as stimulus dynamics, attentional demands, and semantic content), we view these as intrinsic features of real-world communicative contexts. In Experiment 2, we addressed one specific difference by equating stimulus durations across conditions, thereby reducing interpretive ambiguity. Future studies should incorporate more finely matched control conditions to better isolate the effects of communicative intent from other contributing factors such as attention, arousal, and stimulus complexity. Furthermore, it would be interesting to extend the current findings to other relevant crossmodal cues, such as semantic correspondences [30–34] and socio-emotional cues [91].*

Minor:
• Intro, l. 86: “guided by the amount of audiovisual spatial disparity and thus the strength of the common cause prior”. The “thus” sounds a bit misleading, spatial disparity and common cause prior are independent (because the common cause prior is a prior and not the posterior causal probability).

Thank you for highlighting this inaccuracy. We agree with the reviewer and have removed “thus” from the sentence.

• wAV is (I feel) more often called crossmodal bias, and has been introduced (to my knowledge) by Wallace et al. 2004

We opted for the term w_AV_ for reasons of compactness. That said, we acknowledge that the term is not common, albeit used in the past [1]. We are happy to clarify in the manuscript that w_AV_ corresponds to what is often referred to as crossmodal bias:

P. 8 Lines 270-271

*The w_AV_ conceptually corresponds to what is often referred to as crossmodal bias in the literature.*

• L. 405 and Table 1: A probability from a statistical test is never p = 0, but e.g. p < 0.001

Thank you for spotting this mistake. We have substituted “p = 0” with “p < 0.001”.

**References**

1.	Ferrari A, Noppeney U. Attention controls multisensory perception via two distinct mechanisms at different levels of the cortical hierarchy. Summerfield C, editor. PLoS Biol. 2021;19: e3001465. doi:10.1371/journal.pbio.3001465

2.	Provine RR. Yawns, laughs, smiles, tickles, and talking: Naturalistic and laboratory studies of facial action and social communication. Russell, J. A. & Fernández-Dols, J. M. The psychology of facial expression. Russell, J. A. & Fernández-Dols, J. M. Cambridge University Press; 1997. pp. 158–175.

3.	Rohe T, Noppeney U. Cortical Hierarchies Perform Bayesian Causal Inference in Multisensory Perception. Kayser C, editor. PLoS Biol. 2015;13: e1002073. doi:10.1371/journal.pbio.1002073

4.	Acerbi L, Dokka K, Angelaki DE, Ma WJ. Bayesian comparison of explicit and implicit causal inference strategies in multisensory heading perception. Gershman SJ, editor. PLoS Comput Biol. 2018;14: e1006110. doi:10.1371/journal.pcbi.1006110

5.	Senju A, Hasegawa T. Direct gaze captures visuospatial attention. Visual Cognition. 2005;12: 127–144. doi:10.1080/13506280444000157

6.	Barzy M, Morgan R, Cook R, Gray KL. Are social interactions preferentially attended in real-world scenes? Evidence from change blindness. Quarterly Journal of Experimental Psychology. 2023;76: 2293–2302. doi:10.1177/17470218231161044

7.	Skripkauskaite S, Mihai I, Koldewyn K. Attentional bias towards social interactions during viewing of naturalistic scenes. Quarterly Journal of Experimental Psychology. 2023;76: 2303–2311. doi:10.1177/17470218221140879

8.	Williams EH, Cristino F, Cross ES. Human body motion captures visual attention and elicits pupillary dilation. Cognition. 2019;193: 104029. doi:10.1016/j.cognition.2019.104029

9.	Böckler A, Van Der Wel RPRD, Welsh TN. Catching Eyes: Effects of Social and Nonsocial Cues on Attention Capture. Psychol Sci. 2014;25: 720–727. doi:10.1177/0956797613516147

10.	Goupil N, Rayson H, Serraille É, Massera A, Ferrari PF, Hochmann J-R, et al. Visual Preference for Socially Relevant Spatial Relations in Humans and Monkeys. Psychological Science. 2024;35: 681–693.

11.	Li B, Hu W, Hunt A, Sui J. Self-related objects increase alertness and orient attention through top-down saliency. Atten Percept Psychophys. 2022;84: 408–417. doi:10.3758/s13414-021-02429-4

12.	Scheller M, Sui J. Social relevance modulates multisensory integration. Journal of Experimental Psychology: Human Perception and Performance. 2022;48: 1022–1038. doi:10.1037/xhp0001013

13.	Scheller M, Fang H, Sui J. Self as a prior: The malleability of Bayesian multisensory integration to social salience. British J of Psychology. 2024;115: 185–205. doi:10.1111/bjop.12683

14.	Lee JL, Denison R, Ma WJ. Challenging the fixed-criterion model of perceptual decision-making. Neuroscience of Consciousness. 2023;2023: niad010. doi:10.1093/nc/niad010
