# Supplementary material for: Prior expectations guide multisensory integration during face-to-face communication
Source: PLoS Comput Biol. 2025 Sep 12;21(9):e1013468. doi: 10.1371/journal.pcbi.1013468 (PMC12448992; doi:10.1371/journal.pcbi.1013468)
Supplement: Attachment — Submitted filename: Mazzi_Ferrari_response_reviewers_rev2.docx [file pcbi.1013468.s017.docx]

**Response to reviewers**

Thank you for the positive and constructive feedback. We have revised the manuscript thoroughly and responded to each point to the best of our ability. We hope that our changes and clarifications effectively address the remaining concerns. Below, we have attached a copy of the reviews, along with a point-by-point response to each issue raised; our replies are in blue. All additions to the manuscript are explained here, along with the corresponding page numbers. Additionally, all changes are highlighted in the manuscript using track changes.

**Reviewer #3:**

Overall, the authors adress my concerns very well, and their clarifications and additional analyses strengthen the manuscript. My major remaining questions pertains to the comparison of the BCI model to the stochastic fusion and fixed criterion models.

We sincerely thank the reviewer for their positive assessment of the revised manuscript, as well as for their thoughtful feedback. We believe the revisions undertaken in response have significantly enhanced the clarity, interpretability and relevance of our study, as detailed below.

• The authors show that their auditory spatial stimuli work as intended, using generic HRTFs from a database. Still, I don’t understand which of the HRTFs was ‘relevant’ and used in the study (e.g. averaged HRTF, matched HRTFs)? Absolute detail, but important for replication purposes.

Thank you for pointing out the need to clarify the use of HRTFs. We have added the relevant details to the manuscript and referred to the paper that explains how the HRTFs were implemented [1].

P. 6 Lines 168-175

*The auditory stimuli were convolved with spatially selective head-related transfer functions (HRTFs) derived from the ARI HRTF Database [76]. Specifically, stimuli were generated using the “interpolateHRTF” function from the MATLAB Audio Toolbox (documentation: https://it.mathworks.com/help/audio/ref/interpolatehrtf.html). The function utilises a representative subject from the ARI Database and performs spatial interpolation of the Head-Related Impulse Responses (HRIRs) using a Vector Base Amplitude Panning (VBAP) algorithm [77]. For each desired position, the algorithm identifies the three nearest positions in the dataset, and linearly combines the three corresponding HRIRs using the VBAP-based weights.*

• Confounds in control condition: The authors now acklowledge and discuss that the visual static control condition differs in many low- and high-level aspects from the experimental condition. I agree with the authors that ‘communicate intent’ is probably a high-level concept that varies on more than a single sensory dimension or quality when implementing experimentally with some ecological validity. So, it is exactly the usual dilemma between internal and external/ecological valdity. Still, I think better control conditions are conceivable (e.g. dynamic videos with unknown language and gaze diverted from the participant). However, the authors should acknowledge this choice (or limitation) throughout the manuscript, e.g. when motivation the stimuli (demonstrating that it is feature and not a bug).

We thank the reviewer for the thoughtful comment. We now explicitly motivate our choices throughout the manuscript:

P. 6 Lines 161-166 (Stimuli section)

*Collectively, we introduced relevant linguistic content and bodily signals in the communicative condition to maximize its difference from the non-communicative condition, thereby establishing a clear proof-of-concept for the influence of communicative intent on audiovisual causal inference. While this approach introduces both low- and high-level differences between conditions (such as stimulus dynamics, attentional demands, and semantic content), we view these as intrinsic features of real-world communicative contexts.*

P. 7 Lines 220-222 (Design and procedure section)

*As a result, the communicative condition differed from the non-communicative condition in terms of attentional demands and semantic content, as is typically the case in real-world contexts.*

P. 22-23 Lines 734-745 (Discussion section)

*Notably, in the present study, we created pragmatic correspondences by introducing relevant linguistic content and ostensive bodily signals (head and gaze movements). Our design intentionally maximized the contrast between communicative and non-communicative signals to establish a clear proof-of-concept for the influence of communicative intent on audiovisual causal inference. While this approach introduces both low- and high-level differences between conditions (such as stimulus dynamics, attentional demands, and semantic content), we view these as intrinsic features of real-world communicative contexts. In Experiment 2, we addressed one specific difference by equating stimulus durations across conditions, thereby reducing interpretive ambiguity. Future studies should incorporate more finely matched control conditions to better isolate the effects of communicative intent from other contributing factors such as attention, arousal, and stimulus complexity. For example, new control conditions could employ dynamic videos with gaze diverted from the addressee and utterances spoken in an unknown language.*

• The authors justify in length why the stochastic fusion model and fixed criterion models are not suitably for their research question. But this is not very convincing to me, both are plausible and stronger model competitors to BCI. And I think specifically in the context of more complex social signals (compare to the usual flashes and beeps), I would be interesting to learn whether observers adopt non-optimal approximate heuristics. So in this case I think this does not need to be deferred to future studies, but can be implemented in the current study. Even if these models turn to be stronger than BCI, this would not make the study less interesting and important.

We are grateful for the reviewer’s comment, which led us to reconsider the importance of examining the use of non-optimal approximate heuristics.

We compared 8 models in a 4 (architecture: Bayesian Causal Inference vs Fixed Criterion vs Stochastic Fusion vs Forced Fusion) × 2 (action intention conditions: Pooled vs Separated) factorial model space (S4 Fig). In both experiments, Bayesian model comparison favoured the Fixed Criterion Separated model. Focusing on the model parameters, we found that participants exhibited a more liberal criterion k_C_ (i.e. integration response at higher spatial disparities) for the communicative than the non-communicative condition, specifically in Experiment 2. Interestingly, in both experiments, model predictions were highly similar to those obtained with the BCI model (S2 Fig and S3 Fig), and the two model architectures explained the same amount of variance (R^2^ = 0.95 ± 0.01; S4 Table). Moreover, similar to the BCI results, individual increases of w_AV_ for the communicative relative to the non-communicative condition (w_AV_ Com > w_AV_ NCom) positively correlated with the respective effect on the criterion (k_C_ Com > k_C_ NCom) and auditory noise ($\sigma_{A}$ Com > $\sigma_{A}$ NCom) in both experiments (S5 Fig).

The striking similarities between the BCI and Fixed Criterion results made us wonder whether they are inherently capturing the same underlying cognitive mechanism, that is, a stronger a priori belief that signals come from the same source under the communicative condition. If so, there should be a significant correlation between the BCI common cause prior p_common_ and the Fixed Criterion k_C_. Indeed, we found a highly significant correlation in both experiments (Fig. 5). Collectively, these results indicate that observers solve audiovisual causal inference following non-optimal approximate heuristics (i.e. changes in integration criterion) that nevertheless approximate the optimal Bayesian solution with high accuracy. Crucially, a more liberal integration criterion for audiovisual communicative signals may stem from a stronger prior expectation that these signals come from a common cause.

Below, we provide a comprehensive summary of the changes made to the manuscript to incorporate the key insights gained from these analyses, now included in the new section “Assessing non-optimal decision strategies”. We chose this organization to stay true to the original rationale of the study for reasons of clarity and transparency, while also highlighting these important additional analyses and the similarities between BCI and Fixed Criterion.

P. 19-21 Lines 620-691

***Assessing non-optimal decision strategies***

*In our previous analyses, we employed a normative Bayesian framework in which an ideal observer optimally performs perceptual inference. Critically, Forced Fusion offers a relatively weak alternative to Bayesian Causal Inference, as the rigid common-cause assumption is unlikely to hold in the presence of varying spatial disparities. However, models implementing non-optimal decision strategies—such as Fixed Criterion and Stochastic Fusion [55]—offer psychologically plausible, heuristic approximations to perceptual inference that can better accommodate scenarios where observers do not always integrate the signals. Thus, we conducted a follow-up analysis expanding our model space to include Fixed Criterion and Stochastic Fusion models as plausible and stronger model competitors to Bayesian Causal Inference. In the following, we describe the models’ architecture, outline the model comparison approach, and summarize the key findings.*

***Bayesian Modelling***

*The Fixed Criterion and Stochastic Fusion models are equal to Bayesian Causal Inference (see section “General Bayesian modelling approach”) except for how they arbitrate between multisensory integration and segregation.*

*The Fixed Criterion (FC) model exhibits sensitivity to spatial disparity by applying a static disparity threshold to decide whether to integrate or segregate, disregarding the relative sensory uncertainties. Formally:*

$$\hat{S}_{V}= \left( \left| x_{A}- x_{V} \right|\leq k_{C} \right)\hat{S}_{V,C=1}+\left( \left| x_{A}- x_{V} \right|>k_{C} \right)\hat{S}_{V,C=2}$$

$$\hat{S}_{A}= \left( \left| x_{A}- x_{V} \right|\leq k_{C} \right)\hat{S}_{A,C=1}+\left( \left| x_{A}- x_{V} \right|>k_{C} \right)\hat{S}_{A,C=2}$$

*where* $\left| x_{A}- x_{V} \right|$ *is the absolute audiovisual spatial disparity, and k_C_ is the free parameter that represents the fixed criterion for integration (in degrees visual angle). Crucially, k_C_ does not depend on stimulus uncertainty. If auditory information is highly uncertain (as in the case of spatial localisation), a large audiovisual disparity might still plausibly arise from a common source, but the Fixed Criterion model would nonetheless reject it (for more details, [55]). By contrast, if spatial disparity is explainable by noise, BCI may still infer a common cause.*

*The Stochastic Fusion (SF) model exhibits a lack of sensitivity to spatial disparity, similarly to the Forced Fusion model. However, Stochastic Fusion does not mandatorily perform integration. On each trial, it makes a random choice: either it fuses the audiovisual signals or it segregates them. Formally:*

$$\hat{S}_{V}= \left( \eta>0.5 \right)\hat{S}_{V,C=1}+\left( \eta\leq0.5 \right)\hat{S}_{V,C=2}$$

$$\hat{S}_{A}= \left( \eta>0.5 \right)\hat{S}_{A,C=1}+\left( \eta\leq0.5 \right)\hat{S}_{A,C=2}$$

*where η is the free parameter that represents the probability of reporting a common cause. Crucially, η does not depend on spatial disparity or sensory uncertainty. Nonetheless, Stochastic Fusion can partially accommodate scenarios where observers do not always integrate, because it sometimes performs segregation (for more details, [55]). Notably, while Stochastic Fusion incorporates a random decision process, Fixed Criterion leverages a priori information (via k_C_) to guide the decision process, similar to Bayesian Causal Inference (via p_common_).*

*To determine which model architecture best explained each participant’s localisation responses, and to evaluate the modulatory influence of action intention (communicative vs. non-communicative), we performed Bayesian model comparison in a 4 (BCI vs FC vs SF vs FF) × 2 (“Pooled” action intention conditions vs “Separated” conditions) factorial model space (S4a Fig.; see section “General Bayesian modelling approach” for details on model fitting and statistical analyses).*

***Results***

*In both experiments, Bayesian model comparison favoured the Fixed Criterion Separated model (Sb4 Fig.; protected exceedance probability ≈ 1, Bayesian Omnibus Risk < 0.001). Focusing on the model parameters (S4c Fig), we first replicated the effects on the sensory noise parameters. We found no significant difference between the two action intention conditions for the visual noise* $\sigma_{V}$ *(Experiment 1: p = 0.204, es = 0.20, CI(95%) = [-0.02, 0.42], BF_10_ = 0.77; Experiment 2: p = 0.505, es = -0.21, CI(95%) = [-0.58, 0.16], BF_10_ = 0.35), while the auditory noise* $\sigma_{V}$ *was significantly higher in the non-communicative condition in Experiment 1 (p = 0.001, es = -0.83, CI(95%) = [-1.49, -0.16], BF_10_ = 19.95) but not Experiment 2 (p = 0.104, es = 0.46, CI(95%) = [-0.39, 1.31], BF_10_ = 0.55). Crucially, participants exhibited a more liberal criterion k_C_ (i.e. integration response at higher spatial disparities) for the communicative than the non-communicative condition, specifically in Experiment 2 (Experiment 1: p = 0.865, es = 0.22, CI(95%) = [-0.90, 1.35], BF_10_ = 0.19; Experiment 2: p = 0.003, es = 1.77, CI(95%) = [0.67, 2.88], BF_10_ = 165.12; S4 Fig., S2 Table). Interestingly, in both experiments, model predictions were highly similar to those obtained with the BCI model (S2 Fig. and S3 Fig.), and the two model architectures explained the same amount of variance (R^2^ = 0.95 ± 0.01; S4 Table). Moreover, similar to the BCI results, individual increases of w_AV_ for the communicative relative to the non-communicative condition (w_AV_ Com > w_AV_ NCom) positively correlated with the respective effect on the criterion (k_C_ Com > k_C_ NCom) and auditory noise (*$\sigma_{A}$ *Com >* $\sigma_{A}$ *NCom) in both experiments (Experiment 1: k_C_: r = 0.69, p < 0.001;* $\sigma_{A}$*: r = 0.51, p = 0.002; Experiment 2: k_C_: r = 0.78, p < 0.001;* $\sigma_{A}$*: r = 0.35, p = 0.044; S5 Fig.).*

*The striking similarities between the BCI and Fixed Criterion results may suggest that they inherently capture the same underlying cognitive mechanism, that is, a stronger a priori belief that signals come from the same source under the communicative condition. If so, there should be a significant correlation between the BCI common cause prior p_common_ and the Fixed Criterion k_C_. Indeed, we found a highly significant correlation in both experiments (Experiment 1: r = 0.79, p < 0.001; Experiment 2: r = 0.76, p < 0.001; Fig. 5). Collectively, these results indicate that observers solved audiovisual causal inference following non-optimal heuristics (i.e. changes in integration criterion) that nevertheless approximate optimal Bayesian inference with high accuracy. Crucially, a more liberal integration criterion for audiovisual communicative signals likely stems from a stronger prior expectation that these signals come from a common cause.*

Finally, the Abstract, Introduction and Discussion sections have been updated accordingly:

P. 2 Lines 23-28 (Abstract)

*Here, we cast face-to-face communication as a Bayesian Causal Inference problem to formally test whether prior expectations arbitrate between the integration and segregation of vocal and bodily signals. Specifically, we asked whether there is a stronger prior tendency to integrate audiovisual signals that convey the same communicative intention, thus establishing a crossmodal pragmatic correspondence. Additionally, we evaluated whether observers solve causal inference by adopting optimal Bayesian decision strategies or non-optimal approximate heuristics.*

P. 2 Lines 34-39 (Abstract)

*Combining psychophysics with computational modelling, we show that observers solved audiovisual causal inference using non-optimal heuristics that nevertheless approximate optimal Bayesian inference with high accuracy. Remarkably, participants showed a stronger tendency to integrate vocal and bodily information when signals conveyed congruent communicative intent, suggesting that pragmatic correspondences enhance multisensory integration.*

P. 4 Lines 106-114 (Introduction)

*Consequently, several key questions remain unanswered. First, which computational principles govern multisensory integration during face-to-face communication? Second, is integration stronger for communicative than non-communicative signals? If so, why? On the one hand, head and gaze movements towards the observer may attract more attention than looking away, decrease the noise (i.e. increase the reliability) of the attended visual information, and thereby increase its weight in the fusion process under both Forced Fusion and Bayesian Causal Inference accounts [73]. On the other hand, crossmodal pragmatic correspondences among communicative signals may reinforce the prior belief that these signals come from a common cause, as uniquely captured by Bayesian Causal Inference. Critically, observers may also adopt non-optimal heuristic approximations to causal inference [55].*

P. 5 Lines 123-126 (Introduction)

*Our findings demonstrate that multisensory integration was stronger for communicative than non-communicative signals and suggest that pragmatic correspondences strengthened the a priori assumption that multisensory signals belong together. Interestingly, observers’ behaviour was best captured by non-optimal decision strategies that nevertheless approximate optimal Bayesian inference with high accuracy.*

P. 22 Lines 711-712 (Discussion)

*In a first analysis, we contrasted two normative accounts for how we parse the conversational scene into coherent multisensory units while segregating concurrent, yet unrelated, signals.*

P. 23 Lines 755-770 (Discussion)

*In a follow-up analysis, we expanded the model space to include two non-optimal decision strategies offering psychologically plausible, heuristic approximations to perceptual inference [55]. First, the Fixed Criterion model applied a spatial disparity threshold to decide whether to integrate or segregate the audiovisual signals. Second, the Stochastic Fusion model randomly selected between integration and segregation on each trial. In both experiments, Fixed Criterion outperformed competing models in explaining participants’ behaviour, although model predictions were highly similar to those produced by Bayesian Causal Inference. Crucially, participants exhibited a more liberal criterion (i.e. integration response at higher spatial disparities) when vocal and bodily inputs signalled the same communicative intention, as opposed to when this pragmatic correspondence was absent. Interestingly, this effect strongly correlated with an increase in the BCI common cause prior, suggesting that a more liberal integration criterion for audiovisual communicative signals may stem from a stronger prior expectation that these signals come from a common cause. Collectively, these results indicate that, at least in the presence of complex social stimuli, observers solve audiovisual causal inference following non-optimal heuristics (i.e. changes in integration criterion) that nevertheless approximate optimal Bayesian inference with high accuracy. Most importantly, the two computational strategies likely capture the same underlying cognitive mechanism: a stronger prior tendency to integrate vocal and bodily information when signals convey congruent communicative intent.*

**References**

1. Pulkki V. Virtual sound source positioning using vector base amplitude panning. J Audio Eng Soc. 1997;45(6):456–466.
